# dbLGL: an online leukemia gene and literature database for the retrospective comparison of adult and childhood leukemia genetics with literature evidence

**DOI:** 10.1093/database/bay062

**Published:** 2018-06-21

**Authors:** Yining Liu, Mingyu Luo, Zhaochen Jin, Min Zhao, Hong Qu

**Affiliations:** 1 The School of Public Health, Institute for Chemical Carcinogenesis, Guangzhou Medical University, Guangzhou, China; 2 School of Engineering, Faculty of Science, Health, Education and Engineering, University of the Sunshine Coast, Maroochydore DC, Queensland, Australia; 3 Center for Bioinformatics, State Key Laboratory of Protein and Plant Gene Research, College of Life Sciences, Peking University, Beijing, P.R. China

## Abstract

Leukemia is a group of cancers with increased numbers of immature or abnormal leucocytes that originated in the bone marrow and other blood-forming organs. The development of differentially diagnostic biomarkers for different subtypes largely depends on understanding the biological pathways and regulatory mechanisms associated with leukemia-implicated genes. Unfortunately, the leukemia-implicated genes that have been identified thus far are scattered among thousands of published studies, and no systematic summary of the differences between adult and childhood leukemia exists with regard to the causative genetic mutations and genetic mechanisms of the various subtypes. In this study, we performed a systematic literature review of those susceptibility genes reported in small-scale experiments and built an online gene database containing a total of 1805 leukemia-associated genes, available at http://soft.bioinfo-minzhao.org/lgl/. Our comparison of genes from the four primary subtypes and between adult and childhood cases identified a number of potential genes related to patient survival. These curated genes can satisfy a growing demand for further integrating genomics screening for leukemia-associated low-frequency mutated genes.

Database URL: http://soft.bioinfo-minzhao.org/lgl/

## Introduction

Leukemia is one type of blood cancer developed in the bone marrow and other blood-forming organs (1). Based on a combination of onset speed and cell origin, leukemia can generally be divided into four subtypes: acute lymphoblastic leukemia (ALL), acute myeloid leukemia (AML), chronic lymphocytic leukemia (CLL), and chronic myelogenous leukemia (CML). In addition, adult and childhood leukemias have remarkably different cellular and molecular mechanisms. In adults, the most frequently diagnosed leukemias are AML and ALL. Leukemia is also the most diagnosed cancer in children under 15-years old, and most childhood leukemias are an acute type, with ALL accounting for about 75% of pediatric cases (1). For future investigations on the difference between adult and childhood leukemias, it is critical to identify the common and unique genetic suspects to understand their different onset mechanisms. To date, there is a single literature-based database, LeGenD, which contains 70 genes curated from the literature (http://www.bioinformatics.org/legend/legend.htm). However, the page only includes links to the Online Mendelian Inheritance in Man (OMIM) and geneCard databases and lacks comprehensive annotations such as cell location, DNA binding, DNA structure, gene frequency, protein binding and structure, pathway and various other clinical and biological relevant information. Recent high-throughput technology advanced our knowledge on leukemia substantially, especially for those pediatric types. In order to provide a public genetic resource focusing on leukemia for systematic data mining, we constructed a literature-based resource in this article, the database of leukemia gene literature (dbLGL).

## Data curation and database development

### Curation of leukemia-associated genes

The collection of leukemia-associated genes was conducted by an extensive literature curation based on GeneRif (Gene Reference Into Function), which is a collection of short sentences about gene function generated by the National Center for Biotechnology Information (NCBI) (2). In total, we retrieved 8056 relevant GeneRif entries, which we manually curated to remove any non-leukemia genes and extract the leukemia subtypes. The manual literature curation of reading the sentences included the following steps: (i) check that the NCBI Gene ID and Gene Name matched; (ii) exclude those entries unrelated to leukemia genes; (iii), extract the patient information (adults or children) and leukemia subtypes (AML, ALL, CML, CLL etc.); and iv) map non-human genes to human homologous genes using the NCBI homolog database, discarding those lacking a human homolog.

### Leukemia gene and literature database

**Figure 1.**
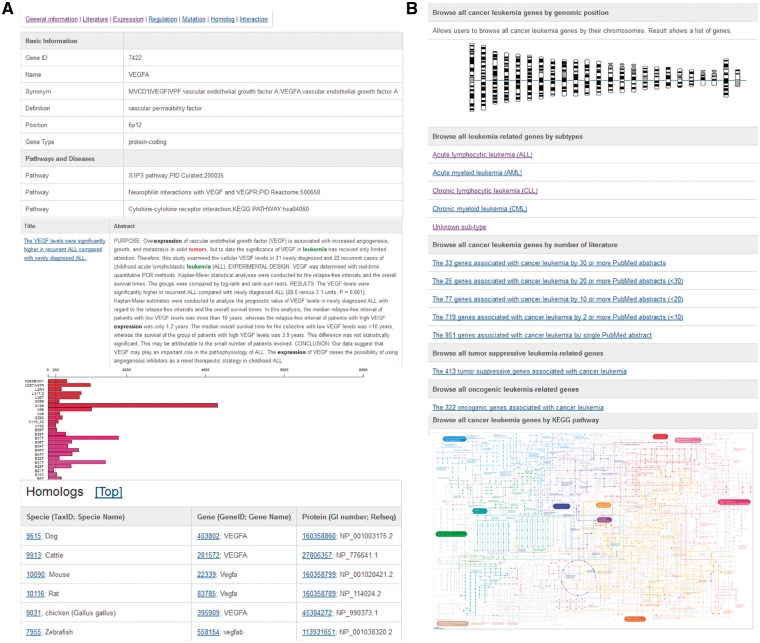
Web interface of the dbLGL. (**A**) A typical gene entry, which includes the basic information, curated literature, gene expression, and pre-computed lncRNA co-expression results using TCGA CRC tumor samples. (**B**) The data browsing interface.

Based on this systematic literature curation of leukemia-associated genes, we developed a user-friendly online database (dbLGL; http://soft.bioinfo-minzhao.org/lgl/) with a browsing function that allows researchers to explore the leukemia subtype-associated genes using chromosome and colored KEGG pathway maps (Figure 1). Homologous sequences from other model species and links to the AnimalTFDB have also been integrated (3). For advanced integrative study, a list of all the leukemia-associated and homologous genes in our database is available for download.

## Data analysis

We obtained a total of 1805 human leukemia-associated genes (Supplementary Table S1). Of these, 1722 genes were derived from adult patients, while only 312 genes were from childhood leukemia patients. In adult leukemia, there were 692, 396, 335 and 389 genes associated with AML, ALL, CML and CLL, respectively. Interestingly, this observation is consistent with AML having the highest incidence rate in adult leukemia patients. Regarding childhood leukemia, over 70% of the genes were relevant to ALL (219 genes), which is also consistent with its higher incidence in childhood. Only 67 human genes were associated with AML (61), CML (4) and CLL (2) in the childhood leukemia cases.

### Biological functional enrichment analysis on the four leukemia subtypes

**Figure 2.**
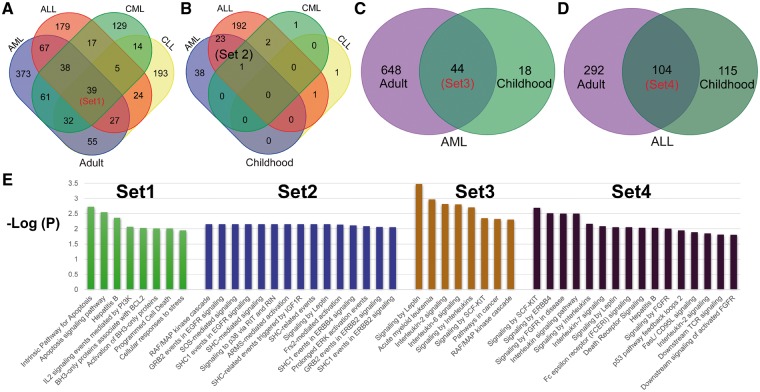
Overlapping and functional enrichment for genes associated with four major leukemia subtypes. (**A**) Venn diagram showing the comparative overlap of the 4 major subtypes in adult patients: ALL, AML, CLL and CML. (**B**) Venn diagram showing the comparative overlap of the four major subtypes in childhood patients. (**C**) The intersection of AML genes from adult and childhood studies. (**D**) The intersection of ALL genes from adult and childhood studies. (**E**) The enriched KEGG pathways for the genes from the four different subtypes.

To provide an overview of the common genetic mechanisms of different leukemia subtypes, we performed a functional enrichment for genes shared by different subtypes (Figure 2). We focused on four gene intersection lists (Figure 2A–D): Set1, 39 genes shared by all four subtypes of adult leukemia; Set2, 24 genes shared by AML and ALL childhood leukemia cases; Set3, 44 AML-related genes present in both adult and child leukemia patients; and Set4, 104 ALL-related genes in both adult and child leukemia patients.

It is not surprising that the common enriched pathways for the four intersection gene lists are mainly related to cell-cycle and cell-proliferation signaling pathways that affect cancer progression. However, there were also distinct functional modules for the four sets. In Set1, there were two pathways related to BH3 proteins (Figure 2E, corrected *P*-values < 0.05), which is a family of typical mitochondrial apoptotic effectors. The BH3 proteins often exercise their function by interactions with *BAK* and other pro-apoptotic proteins. In contrast, the activity of BH3-only proteins is usually inhibited by anti-apoptotic genes from the BH1–4 BCL-2 family (4). For example, as a member of the BH3 family, *PUMA* (p53 up-regulated modulator of apoptosis) is one of the sensors of cellular damage and can inactivate prosurvival Bcl-2 family members, which commits the cell to apoptosis. By acting as a transcriptional target of *TP53*, *PUMA* has elevated expression in CLL cells with normally functioning *TP53* compared to those cells with dysfunctional *TP53* (5). Combined together, these may indicate that *PUMA* may play a role in *TP53*-mediated cell apoptosis (5). The expression of another BH3 gene, *BIM*, can result in conformational changes of *BAK*, which will further interfere with the cell-death signaling pathway regulated by *PI3K*/*AKT* and *MEK1*/*2* (6). Similarly, *NOXA*, a BH3 protein, is activated during apoptosis due to triggering by *M2YN* (7). Given the important roles played by these BH3-family genes, they can be considered leukemia-specific therapeutic targets.

In Set2, we found six significantly enriched SHC-related pathways (corrected *P*-values < 0.05; Figure 2E). In these signaling pathways, SHC-related genes can interact with the signaling molecules *EGFR*, *ERBB2*, *ERBB4* and *IGF1R* by phosphorylation to further activate cell-proliferation and apoptosis-related pathways. For example, *SHC1* phosphorylated by *ERBB4* can further recruit *GRB2* and *SOS1* to activate downstream RAF/MAPK pathways (8). This also explains why GRB2 and SOS1-related pathways were significantly enriched in Set2. More interesting, the RAF/MAPK pathway is usually regulated by upstream regulators such as *RIT1*/*RIN*, *ARMS* and *FRS2*, which are all enriched in the over-represented pathways in Set2. All of these closely related functional modules centered on the SHC genes might provide multiple treatment targets for childhood AML and ALL. By comparing the enriched pathways in Set1 and Set2, we may also gain insight in the differences between the common upstream regulators in the adult and childhood leukemia samples. For example, the common signaling pathways for the four types of adult leukemia are primarily through PI3K/AKT signaling, while the enriched downstream signaling pathways in childhood leukemia are mainly related to MAPK signaling (Figure 2E).

The enrichment analysis of the 44 genes in Set3 identified three interleukin-related regulatory pathways involving IL-2 and IL-6, among others (Figure 2E). These results highlight the critical roles of interleukin in both adult and childhood AML. In essence, the IL-2-dependent cell proliferation, differentiation, and apoptosis processes are fully controlled by the RAS/MAPK and PI3K signaling pathways (9). Interleukin-6 could also initiate multiple downstream regulatory signaling pathways, including JAK/STAT3, RAS/MAPK and PI3K. Similarly, the cytokines IL-3 and IL-5 also have the capacity to activate these three signaling pathways (JAK/STAT, RAS/MAPK and PI3K).

For Set4, we found multiple enriched pathways related to fibroblast growth factor (*FGFR*), which plays a role in hematopoiesis and tumorigenesis by activating the PI3K/AKT pathway to inhibit apoptosis. When compared with Set3, we also found some novel pathways, which may reflect the genetic differences between AML and ALL. For example, the leptin-mediated signaling pathway was over-represented in Set4. Leptin can trigger STAT and MAPK phosphorylation, thereby affecting downstream cellular apoptosis and proliferation (10). In contrast, signaling by stem cell factor signaling pathway (SCF-KIT) was enriched in both Set3 and Set4, which may imply that AML and ALL have some molecular mechanisms in common. SCF-KIT signaling could further regulate downstream leukemia-associated pathways, including MAPK, PI3K and JAK/STAT (11).

### Potential prognostic application

**Figure 3.**
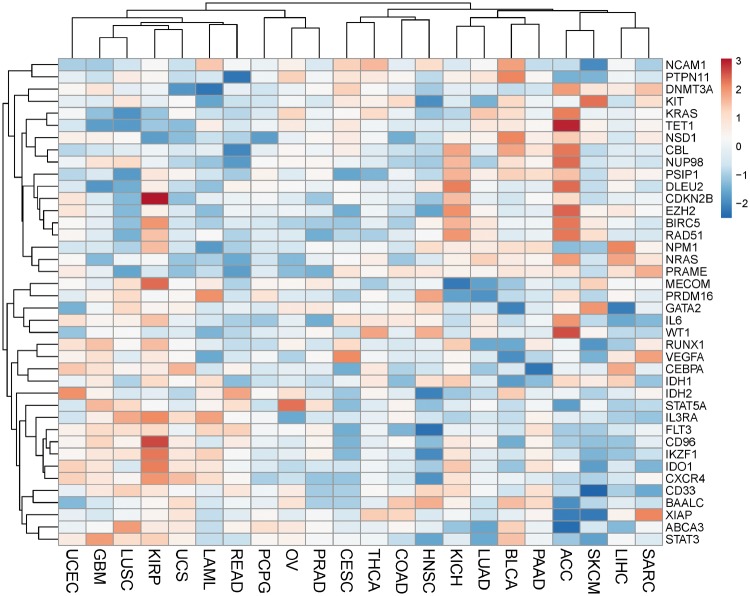
Heatmap of prognostic Z-scores of 40 genes in the 22 TCGA cancer set. The prognostic Z-scores obtained from the PRECOG database are represented by the scale bar. The polarity of the prognostic Z-score reflects the direction of association. TCGA cancer type abbreviations: ACC, Adrenocortical carcinoma; BLCA, bladder urothelial carcinoma; CESC, cervical squamous cell carcinoma and endocervical adenocarcinoma; COAD, colon adenocarcinoma; GBM, glioblastoma multiforme; HNSC, head and Neck squamous cell carcinoma; KICH, kidney chromophobe; KIRP, kidney renal papillary cell carcinoma; LAML, acute myeloid leukemia; LIHC, liver hepatocellular carcinoma; LUAD, lung adenocarcinoma; LUSC, lung squamous cell carcinoma; OV, ovarian serous cystadenocarcinoma; PAAD, pancreatic adenocarcinoma; PCPG, pheochromocytoma and paraganglioma; PRAD, prostate adenocarcinoma; READ, rectum adenocarcinoma; SARC, sarcoma; SKCM, skin cutaneous melanoma; THCA, thyroid carcinoma; UCEC, uterine corpus endometrial carcinoma; UCS, uterine carcinosarcoma.

To further explore potential prognostic applications, we overlapped Set3 with the datasets in prognostic database PRECOG with survival outcomes for the AML cohort (12). The PRECOG database included 22 TCGA datasets generated under unified data quality control and processing procedure. For each gene, PRECOG computed Z-scores to characterize the gene expression feature and clinical outcomes across multiple TCGA cancer types. In general, a positive Z-score for a gene related to a specific cancer type means higher expression (adverse survival), and a negative Z-score reflects lower expression (favorable survival). Of the 44 genes in Set3, 40 are shown in the heatmap of the PRECOG Z-scores for the 22 TCGA cancer types (Figure 3). For example, 3 (*PRDM16*, *IL3RA*, and *IDH1*) of the 40 genes have a prognostic Z-score > 1.96 in the TCGA AML dataset, which is equivalent to a two-tailed *P* < 0.05. In particular, *PRDM16* is a zinc-finger transcription factor that controls muscle cell differentiation (13), and *IL3RA* is an IL-3-specific subunit of a heterodimeric cytokine receptor. Since IL-3 is a multipotent hematopoietic growth factor produced by activated T cells, monocytes/macrophages, and stroma cells, it may be an attractive way to monitor the validation of treatment-related mortality and morbidity in oncology patients (14). We also found one of most important rate-limiting enzymes, *IDH1*, which is often the first driver mutation in the development of diffuse gliomas. Glioblastomas with a wild-type IDH1 gene have a median overall survival of only 1 year, whereas patients with an IDH1-mutated glioblastoma have a median overall survival of over 2 years (15). In conclusion, we found that three genes (*PRDM16, IL3RA* and *IDH1*) are closely related to patient survival in many other cancers, which may provide evidence for the similar mechanism in leukemia.

## Conclusion and discussion

We developed a literature-based knowledge base of leukemia genes with comprehensive annotations. Although leukemia is the most common cancer among children and teens, accounting for almost one in three cancers, the knowledge of the genetic determinants of childhood leukemia is not clear compared with the adult leukemia patients. From our data collection, there are 1722 adult and 312 childhood related genes from the literature. However, only 83 genes are shared between adult and childhood leukemia, which confirms the different molecular mechanisms between adult and pediatric leukemia. Our additional integrative analysis provides the first insight into the heterogeneous genetic structures of different subtypes of leukemia in adults and childhoods, which may have prognostic significance. Our future direction will focus on the childhood leukemia-unique gene set, which may help to understand underlying disease mechanisms and identify novel therapies for childhood leukemia.

## Supplementary data

Supplementary data are available at *Database* Online.

## Funding

This work was supported by the National Natural Science Foundation of China (No. 31671375), the National Key Research and Development Program of China (No. 2017YFC1201200), and the research start-up fellowship of university of sunshine coast to M.Z.

*Conflict of interest*. None declared.

## Supplementary Material

Supplementary Data
