# Supplementary material for: dbLGL: an online leukemia gene and literature database for the retrospective comparison of adult and childhood leukemia genetics with literature evidence
Source: Database (Oxford). 2018 Jun 21;2018:bay062. doi: 10.1093/database/bay062 (PMC6014132; doi:10.1093/database/bay062)
Supplement: Supplementary Data [file bay062_supp_st_1.doc]

| **GeneID** | **GeneSymbol** | **Cytoband** | **FullName** | **GeneType** |
| --- | --- | --- | --- | --- |
| 10 | NAT2 | 8p22 | N-acetyltransferase 2 | protein-coding |
| 1000 | CDH2 | 18q12.1 | cadherin 2 | protein-coding |
| 10000 | AKT3 | 1q43-q44 | AKT serine/threonine kinase 3 | protein-coding |
| 100048912 | CDKN2B-AS1 | 9p21.3 | CDKN2B antisense RNA 1 | ncRNA |
| 10006 | ABI1 | 10p12.1 | abl interactor 1 | protein-coding |
| 100124700 | HOTAIR | 12q13.13 | HOX transcript antisense RNA | ncRNA |
| 100126333 | MIR708 | 11q14.1 | microRNA 708 | ncRNA |
| 10013 | HDAC6 | Xp11.23 | histone deacetylase 6 | protein-coding |
| 100131234 | MIR181A1HG | 1q32.1 | MIR181A1 host gene | ncRNA |
| 100132285 | KIR2DS2 | 19q13.4 | killer cell immunoglobulin like receptor, two Ig domains and short cytoplasmic tail 2 | protein-coding |
| 100133941 | CD24 | 6q21 | CD24 molecule | protein-coding |
| 10017 | BCL2L10 | 15q21.2 | BCL2 like 10 | protein-coding |
| 10018 | BCL2L11 | 2q13 | BCL2 like 11 | protein-coding |
| 10019 | SH2B3 | 12q24.12 | SH2B adaptor protein 3 | protein-coding |
| 1003 | CDH5 | 16q21 | cadherin 5 | protein-coding |
| 100302219 | MIR1245A | 2q32.2 | microRNA 1245a | ncRNA |
| 100507436 | MICA | 6p21.33 | MHC class I polypeptide-related sequence A | protein-coding |
| 10051 | SMC4 | 3q25.33 | structural maintenance of chromosomes 4 | protein-coding |
| 100532731 | COMMD3-BMI1 | 10p12.2 | COMMD3-BMI1 readthrough | protein-coding |
| 10058 | ABCB6 | 2q35 | ATP binding cassette subfamily B member 6 (Langereis blood group) | protein-coding |
| 10081 | PDCD7 | 15q22.31 | programmed cell death 7 | protein-coding |
| 10111 | RAD50 | 5q31.1 | RAD50 double strand break repair protein | protein-coding |
| 1012 | CDH13 | 16q23.3 | cadherin 13 | protein-coding |
| 10128 | LRPPRC | 2p21 | leucine rich pentatricopeptide repeat containing | protein-coding |
| 10130 | PDIA6 | 2p25.1 | protein disulfide isomerase family A member 6 | protein-coding |
| 10152 | ABI2 | 2q33.2 | abl interactor 2 | protein-coding |
| 10153 | CEBPZ | 2p22.2 | CCAAT/enhancer binding protein zeta | protein-coding |
| 1017 | CDK2 | 12q13.2 | cyclin dependent kinase 2 | protein-coding |
| 10180 | RBM6 | 3p21.31 | RNA binding motif protein 6 | protein-coding |
| 1019 | CDK4 | 12q14.1 | cyclin dependent kinase 4 | protein-coding |
| 10197 | PSME3 | 17q21.31 | proteasome activator subunit 3 | protein-coding |
| 1020 | CDK5 | 7q36.1 | cyclin dependent kinase 5 | protein-coding |
| 1021 | CDK6 | 7q21.2 | cyclin dependent kinase 6 | protein-coding |
| 10215 | OLIG2 | 21q22.11 | oligodendrocyte lineage transcription factor 2 | protein-coding |
| 10217 | CTDSPL | 3p22.2 | CTD small phosphatase like | protein-coding |
| 10219 | KLRG1 | 12p13.31 | killer cell lectin like receptor G1 | protein-coding |
| 1022 | CDK7 | 5q13.2 | cyclin dependent kinase 7 | protein-coding |
| 10221 | TRIB1 | 8q24.13 | tribbles pseudokinase 1 | protein-coding |
| 10225 | CD96 | 3q13.13-q13.2 | CD96 molecule | protein-coding |
| 10232 | MSLN | 16p13.3 | mesothelin | protein-coding |
| 1025 | CDK9 | 9q34.11 | cyclin dependent kinase 9 | protein-coding |
| 10257 | ABCC4 | 13q32.1 | ATP binding cassette subfamily C member 4 | protein-coding |
| 1026 | CDKN1A | 6p21.2 | cyclin dependent kinase inhibitor 1A | protein-coding |
| 1027 | CDKN1B | 12p13.1 | cyclin dependent kinase inhibitor 1B | protein-coding |
| 102723996 | LOC102723996 | 21p12 | ICOS ligand | protein-coding |
| 10274 | STAG1 | 3q22.3 | stromal antigen 1 | protein-coding |
| 1028 | CDKN1C | 11p15.4 | cyclin dependent kinase inhibitor 1C | protein-coding |
| 10288 | LILRB2 | 19q13.42 | leukocyte immunoglobulin like receptor B2 | protein-coding |
| 1029 | CDKN2A | 9p21.3 | cyclin dependent kinase inhibitor 2A | protein-coding |
| 103 | ADAR | 1q21.3 | adenosine deaminase, RNA specific | protein-coding |
| 1030 | CDKN2B | 9p21.3 | cyclin dependent kinase inhibitor 2B | protein-coding |
| 10301 | DLEU1 | 13q14.2-q14.3 | deleted in lymphocytic leukemia 1 | ncRNA |
| 1032 | CDKN2D | 19p13.2 | cyclin dependent kinase inhibitor 2D | protein-coding |
| 10320 | IKZF1 | 7p12.2 | IKAROS family zinc finger 1 | protein-coding |
| 10333 | TLR6 | 4p14 | toll like receptor 6 | protein-coding |
| 10365 | KLF2 | 19p13.11 | Kruppel like factor 2 | protein-coding |
| 10370 | CITED2 | 6q24.1 | Cbp/p300 interacting transactivator with Glu/Asp rich carboxy-terminal domain 2 | protein-coding |
| 10397 | NDRG1 | 8q24.22 | N-myc downstream regulated 1 | protein-coding |
| 10401 | PIAS3 | 1q21.1 | protein inhibitor of activated STAT 3 | protein-coding |
| 10413 | YAP1 | 11q22.1 | Yes associated protein 1 | protein-coding |
| 1043 | CD52 | 1p36.11 | CD52 molecule | protein-coding |
| 10434 | LYPLA1 | 8q11.23 | lysophospholipase I | protein-coding |
| 1045 | CDX2 | 13q12.2 | caudal type homeobox 2 | protein-coding |
| 1046 | CDX4 | Xq13.2 | caudal type homeobox 4 | protein-coding |
| 10461 | MERTK | 2q13 | MER proto-oncogene, tyrosine kinase | protein-coding |
| 10499 | NCOA2 | 8q13.3 | nuclear receptor coactivator 2 | protein-coding |
| 1050 | CEBPA | 19q13.11 | CCAAT/enhancer binding protein alpha | protein-coding |
| 10507 | SEMA4D | 9q22.2 | semaphorin 4D | protein-coding |
| 1051 | CEBPB | 20q13.13 | CCAAT/enhancer binding protein beta | protein-coding |
| 1052 | CEBPD | 8q11.21 | CCAAT/enhancer binding protein delta | protein-coding |
| 10529 | NEBL | 10p12.31 | nebulette | protein-coding |
| 1053 | CEBPE | 14q11.2 | CCAAT/enhancer binding protein epsilon | protein-coding |
| 10538 | BATF | 14q24.3 | basic leucine zipper ATF-like transcription factor | protein-coding |
| 1054 | CEBPG | 19q13.11 | CCAAT/enhancer binding protein gamma | protein-coding |
| 10550 | ARL6IP5 | 3p14.1 | ADP ribosylation factor like GTPase 6 interacting protein 5 | protein-coding |
| 10555 | AGPAT2 | 9q34.3 | 1-acylglycerol-3-phosphate O-acyltransferase 2 | protein-coding |
| 10560 | SLC19A2 | 1q24.2 | solute carrier family 19 member 2 | protein-coding |
| 10562 | OLFM4 | 13q14.3 | olfactomedin 4 | protein-coding |
| 10563 | CXCL13 | 4q21.1 | C-X-C motif chemokine ligand 13 | protein-coding |
| 10578 | GNLY | 2p11.2 | granulysin | protein-coding |
| 10592 | SMC2 | 9q31.1 | structural maintenance of chromosomes 2 | protein-coding |
| 10599 | SLCO1B1 | 12p12.1 | solute carrier organic anion transporter family member 1B1 | protein-coding |
| 10600 | USP16 | 21q21.3 | ubiquitin specific peptidase 16 | protein-coding |
| 10628 | TXNIP | 1q21.1 | thioredoxin interacting protein | protein-coding |
| 10642 | IGF2BP1 | 17q21.32 | insulin like growth factor 2 mRNA binding protein 1 | protein-coding |
| 10643 | IGF2BP3 | 7p15.3 | insulin like growth factor 2 mRNA binding protein 3 | protein-coding |
| 10661 | KLF1 | 19p13.13 | Kruppel like factor 1 | protein-coding |
| 10664 | CTCF | 16q22.1 | CCCTC-binding factor | protein-coding |
| 10666 | CD226 | 18q22.2 | CD226 molecule | protein-coding |
| 10673 | TNFSF13B | 13q33.3 | tumor necrosis factor superfamily member 13b | protein-coding |
| 10735 | STAG2 | Xq25 | stromal antigen 2 | protein-coding |
| 10769 | PLK2 | 5q11.2 | polo like kinase 2 | protein-coding |
| 10800 | CYSLTR1 | Xq21.1 | cysteinyl leukotriene receptor 1 | protein-coding |
| 10801 | Sep-09 | 17q25.3 | septin 9 | protein-coding |
| 10803 | CCR9 | 3p21.31 | C-C motif chemokine receptor 9 | protein-coding |
| 10850 | CCL27 | 9p13.3 | C-C motif chemokine ligand 27 | protein-coding |
| 10855 | HPSE | 4q21.23 | heparanase | protein-coding |
| 10859 | LILRB1 | 19q13.42 | leukocyte immunoglobulin like receptor B1 | protein-coding |
| 10863 | ADAM28 | 8p21.2 | ADAM metallopeptidase domain 28 | protein-coding |
| 10870 | HCST | 19q13.12 | hematopoietic cell signal transducer | protein-coding |
| 10874 | NMU | 4q12 | neuromedin U | protein-coding |
| 10919 | EHMT2 | 6p21.33 | euchromatic histone lysine methyltransferase 2 | protein-coding |
| 10928 | RALBP1 | 18p11.22 | ralA binding protein 1 | protein-coding |
| 10962 | MLLT11 | 1q21.3 | myeloid/lymphoid or mixed-lineage leukemia; translocated to, 11 | protein-coding |
| 10979 | FERMT2 | 14q22.1 | fermitin family member 2 | protein-coding |
| 10987 | COPS5 | 8q13.1 | COP9 signalosome subunit 5 | protein-coding |
| 11006 | LILRB4 | 19q13.42 | leukocyte immunoglobulin like receptor B4 | protein-coding |
| 11009 | IL24 | 1q32.1 | interleukin 24 | protein-coding |
| 11010 | GLIPR1 | 12q21.2 | GLI pathogenesis related 1 | protein-coding |
| 11035 | RIPK3 | 14q12 | receptor interacting serine/threonine kinase 3 | protein-coding |
| 11040 | PIM2 | Xp11.23 | Pim-2 proto-oncogene, serine/threonine kinase | protein-coding |
| 11047 | ADRM1 | 20q13.33 | adhesion regulating molecule 1 | protein-coding |
| 1106 | CHD2 | 15q26.1 | chromodomain helicase DNA binding protein 2 | protein-coding |
| 11091 | WDR5 | 9q34.2 | WD repeat domain 5 | protein-coding |
| 1111 | CHEK1 | 11q24.2 | checkpoint kinase 1 | protein-coding |
| 11126 | CD160 | 1q21.1 | CD160 molecule | protein-coding |
| 11151 | CORO1A | 16p11.2 | coronin 1A | protein-coding |
| 11156 | PTP4A3 | 8q24.3 | protein tyrosine phosphatase type IVA, member 3 | protein-coding |
| 11168 | PSIP1 | 9p22.3 | PC4 and SFRS1 interacting protein 1 | protein-coding |
| 1118 | CHIT1 | 1q32.1 | chitinase 1 | protein-coding |
| 11184 | MAP4K1 | 19q13.2 | mitogen-activated protein kinase kinase kinase kinase 1 | protein-coding |
| 11197 | WIF1 | 12q14.3 | WNT inhibitory factor 1 | protein-coding |
| 11200 | CHEK2 | 22q12.1 | checkpoint kinase 2 | protein-coding |
| 11214 | AKAP13 | 15q25.3 | A-kinase anchoring protein 13 | protein-coding |
| 112939 | NACC1 | 19p13.13 | nucleus accumbens associated 1 | protein-coding |
| 113235 | SLC46A1 | 17q11.2 | solute carrier family 46 member 1 | protein-coding |
| 11331 | PHB2 | 12p13.31 | prohibitin 2 | protein-coding |
| 11339 | OIP5 | 15q15.1 | Opa interacting protein 5 | protein-coding |
| 114548 | NLRP3 | 1q44 | NLR family pyrin domain containing 3 | protein-coding |
| 1147 | CHUK | 10q24.31 | conserved helix-loop-helix ubiquitous kinase | protein-coding |
| 115 | ADCY9 | 16p13.3 | adenylate cyclase 9 | protein-coding |
| 115350 | FCRL1 | 1q23.1 | Fc receptor like 1 | protein-coding |
| 115352 | FCRL3 | 1q23.1 | Fc receptor like 3 | protein-coding |
| 1154 | CISH | 3p21.2 | cytokine inducible SH2 containing protein | protein-coding |
| 115650 | TNFRSF13C | 22q13.2 | TNF receptor superfamily member 13C | protein-coding |
| 115727 | RASGRP4 | 19q13.2 | RAS guanyl releasing protein 4 | protein-coding |
| 1160 | CKMT2 | 5q14.1 | creatine kinase, mitochondrial 2 | protein-coding |
| 116173 | CMTM5 | 14q11.2 | CKLF like MARVEL transmembrane domain containing 5 | protein-coding |
| 116988 | AGAP3 | 7q36.1 | ArfGAP with GTPase domain, ankyrin repeat and PH domain 3 | protein-coding |
| 117581 | TWIST2 | 2q37.3 | twist family bHLH transcription factor 2 | protein-coding |
| 1178 | CLC | 19q13.2 | Charcot-Leyden crystal galectin | protein-coding |
| 119391 | GSTO2 | 10q25.1 | glutathione S-transferase omega 2 | protein-coding |
| 121504 | HIST4H4 | 12p12.3 | histone cluster 4 H4 | protein-coding |
| 121599 | SPIC | 12q23.2 | Spi-C transcription factor | protein-coding |
| 122953 | JDP2 | 14q24.3 | Jun dimerization protein 2 | protein-coding |
| 123169 | LEO1 | 15q21.2 | LEO1 homolog, Paf1/RNA polymerase II complex component | protein-coding |
| 1233 | CCR4 | 3p22.3 | C-C motif chemokine receptor 4 | protein-coding |
| 1234 | CCR5 | 3p21.31 | C-C motif chemokine receptor 5 (gene/pseudogene) | protein-coding |
| 1236 | CCR7 | 17q21.2 | C-C motif chemokine receptor 7 | protein-coding |
| 1241 | LTB4R | 14q12 | leukotriene B4 receptor | protein-coding |
| 1244 | ABCC2 | 10q24.2 | ATP binding cassette subfamily C member 2 | protein-coding |
| 124540 | MSI2 | 17q22 | musashi RNA binding protein 2 | protein-coding |
| 1269 | CNR2 | 1p36.11 | cannabinoid receptor 2 | protein-coding |
| 127086 | XRCC6P3 | 1q41 | X-ray repair cross complementing 6 pseudogene 3 | pseudo |
| 127933 | UHMK1 | 1q23.3 | U2AF homology motif kinase 1 | protein-coding |
| 128710 | SLX4IP | 20p12.2 | SLX4 interacting protein | protein-coding |
| 131450 | CD200R1 | 3q13.2 | CD200 receptor 1 | protein-coding |
| 1316 | KLF6 | 10p15.2 | Kruppel like factor 6 | protein-coding |
| 1326 | MAP3K8 | 10p11.23 | mitogen-activated protein kinase kinase kinase 8 | protein-coding |
| 133396 | IL31RA | 5q11.2 | interleukin 31 receptor A | protein-coding |
| 135138 | PACRG | 6q26 | PARK2 coregulated | protein-coding |
| 137196 | CCDC26 | 8q24.21 | CCDC26 long non-coding RNA | ncRNA |
| 1380 | CR2 | 1q32.2 | complement C3d receptor 2 | protein-coding |
| 1385 | CREB1 | 2q33.3 | cAMP responsive element binding protein 1 | protein-coding |
| 1387 | CREBBP | 16p13.3 | CREB binding protein | protein-coding |
| 1389 | CREBL2 | 12p13.1 | cAMP responsive element binding protein like 2 | protein-coding |
| 1390 | CREM | 10p11.21 | cAMP responsive element modulator | protein-coding |
| 139285 | AMER1 | Xq11.2 | APC membrane recruitment protein 1 | protein-coding |
| 1394 | CRHR1 | 17q21.31 | corticotropin releasing hormone receptor 1 | protein-coding |
| 1398 | CRK | 17p13.3 | CRK proto-oncogene, adaptor protein | protein-coding |
| 1399 | CRKL | 22q11.21 | CRK like proto-oncogene, adaptor protein | protein-coding |
| 140688 | NOL4L | 20q11.21 | nucleolar protein 4 like | protein-coding |
| 1407 | CRY1 | 12q23.3 | cryptochrome circadian clock 1 | protein-coding |
| 140885 | SIRPA | 20p13 | signal regulatory protein alpha | protein-coding |
| 142 | PARP1 | 1q42.12 | poly(ADP-ribose) polymerase 1 | protein-coding |
| 1429 | CRYZ | 1p31.1 | crystallin zeta | protein-coding |
| 1432 | MAPK14 | 6p21.31 | mitogen-activated protein kinase 14 | protein-coding |
| 1436 | CSF1R | 5q32 | colony stimulating factor 1 receptor | protein-coding |
| 143686 | SESN3 | 11q21 | sestrin 3 | protein-coding |
| 1437 | CSF2 | 5q31.1 | colony stimulating factor 2 | protein-coding |
| 1438 | CSF2RA | Xp22.33 and Yp11.2 | colony stimulating factor 2 receptor alpha subunit | protein-coding |
| 1439 | CSF2RB | 22q12.3 | colony stimulating factor 2 receptor beta common subunit | protein-coding |
| 1440 | CSF3 | 17q21.1 | colony stimulating factor 3 | protein-coding |
| 1441 | CSF3R | 1p34.3 | colony stimulating factor 3 receptor | protein-coding |
| 144455 | E2F7 | 12q21.2 | E2F transcription factor 7 | protein-coding |
| 1445 | CSK | 15q24.1 | c-src tyrosine kinase | protein-coding |
| 1457 | CSNK2A1 | 20p13 | casein kinase 2 alpha 1 | protein-coding |
| 1460 | CSNK2B | 6p21.33 | casein kinase 2 beta | protein-coding |
| 1464 | CSPG4 | 15q24.2 | chondroitin sulfate proteoglycan 4 | protein-coding |
| 146852 | ODF4 | 17p13.1 | outer dense fiber of sperm tails 4 | protein-coding |
| 1482 | NKX2-5 | 5q35.1 | NK2 homeobox 5 | protein-coding |
| 1485 | CTAG1B | Xq28 | cancer/testis antigen 1B | protein-coding |
| 1487 | CTBP1 | 4p16.3 | C-terminal binding protein 1 | protein-coding |
| 1488 | CTBP2 | 10q26.13 | C-terminal binding protein 2 | protein-coding |
| 1490 | CTGF | 6q23.2 | connective tissue growth factor | protein-coding |
| 1493 | CTLA4 | 2q33.2 | cytotoxic T-lymphocyte associated protein 4 | protein-coding |
| 1495 | CTNNA1 | 5q31.2 | catenin alpha 1 | protein-coding |
| 1499 | CTNNB1 | 3p22.1 | catenin beta 1 | protein-coding |
| 1508 | CTSB | 8p23.1 | cathepsin B | protein-coding |
| 1509 | CTSD | 11p15.5 | cathepsin D | protein-coding |
| 151011 | Sep-10 | 2q13 | septin 10 | protein-coding |
| 1511 | CTSG | 14q12 | cathepsin G | protein-coding |
| 1515 | CTSV | 9q22.33 | cathepsin V | protein-coding |
| 151888 | BTLA | 3q13.2 | B and T lymphocyte associated | protein-coding |
| 152137 | CCDC50 | 3q28 | coiled-coil domain containing 50 | protein-coding |
| 1523 | CUX1 | 7q22.1 | cut like homeobox 1 | protein-coding |
| 1524 | CX3CR1 | 3p22.2 | C-X3-C motif chemokine receptor 1 | protein-coding |
| 153 | ADRB1 | 10q25.3 | adrenoceptor beta 1 | protein-coding |
| 1535 | CYBA | 16q24.2 | cytochrome b-245 alpha chain | protein-coding |
| 154 | ADRB2 | 5q32 | adrenoceptor beta 2 | protein-coding |
| 1540 | CYLD | 16q12.1 | CYLD lysine 63 deubiquitinase | protein-coding |
| 1543 | CYP1A1 | 15q24.1 | cytochrome P450 family 1 subfamily A member 1 | protein-coding |
| 1544 | CYP1A2 | 15q24.1 | cytochrome P450 family 1 subfamily A member 2 | protein-coding |
| 1545 | CYP1B1 | 2p22.2 | cytochrome P450 family 1 subfamily B member 1 | protein-coding |
| 155 | ADRB3 | 8p11.23 | adrenoceptor beta 3 | protein-coding |
| 1555 | CYP2B6 | 19q13.2 | cytochrome P450 family 2 subfamily B member 6 | protein-coding |
| 1565 | CYP2D6 | 22q13.2 | cytochrome P450 family 2 subfamily D member 6 | protein-coding |
| 1571 | CYP2E1 | 10q26.3 | cytochrome P450 family 2 subfamily E member 1 | protein-coding |
| 1577 | CYP3A5 | 7q22.1 | cytochrome P450 family 3 subfamily A member 5 | protein-coding |
| 158158 | RASEF | 9q21.32 | RAS and EF-hand domain containing | protein-coding |
| 1588 | CYP19A1 | 15q21.2 | cytochrome P450 family 19 subfamily A member 1 | protein-coding |
| 160364 | CLEC12A | 12p13.31 | C-type lectin domain family 12 member A | protein-coding |
| 160365 | CLECL1 | 12p13.31 | C-type lectin like 1 | protein-coding |
| 160728 | SLC5A8 | 12q23.1-q23.2 | solute carrier family 5 member 8 | protein-coding |
| 1612 | DAPK1 | 9q21.33 | death associated protein kinase 1 | protein-coding |
| 1616 | DAXX | 6p21.32 | death domain associated protein | protein-coding |
| 161742 | SPRED1 | 15q14 | sprouty related EVH1 domain containing 1 | protein-coding |
| 1627 | DBN1 | 5q35.3 | drebrin 1 | protein-coding |
| 162979 | ZNF296 | 19q13.32 | zinc finger protein 296 | protein-coding |
| 1630 | DCC | 18q21.2 | DCC netrin 1 receptor | protein-coding |
| 1633 | DCK | 4q13.3 | deoxycytidine kinase | protein-coding |
| 1634 | DCN | 12q21.33 | decorin | protein-coding |
| 1636 | ACE | 17q23.3 | angiotensin I converting enzyme | protein-coding |
| 1641 | DCX | Xq23 | doublecortin | protein-coding |
| 1647 | GADD45A | 1p31.3 | growth arrest and DNA damage inducible alpha | protein-coding |
| 1649 | DDIT3 | 12q13.3 | DNA damage inducible transcript 3 | protein-coding |
| 1654 | DDX3X | Xp11.4 | DEAD-box helicase 3, X-linked | protein-coding |
| 1655 | DDX5 | 17q23.3 | DEAD-box helicase 5 | protein-coding |
| 166 | AES | 19p13.3 | amino-terminal enhancer of split | protein-coding |
| 1667 | DEFA1 | 8p23.1 | defensin alpha 1 | protein-coding |
| 1668 | DEFA3 | 8p23.1 | defensin alpha 3 | protein-coding |
| 171017 | ZNF384 | 12p13.31 | zinc finger protein 384 | protein-coding |
| 171023 | ASXL1 | 20q11.21 | additional sex combs like 1, transcriptional regulator | protein-coding |
| 171558 | PTCRA | 6p21.1 | pre T-cell antigen receptor alpha | protein-coding |
| 1719 | DHFR | 5q14.1 | dihydrofolate reductase | protein-coding |
| 1728 | NQO1 | 16q22.1 | NAD(P)H quinone dehydrogenase 1 | protein-coding |
| 1747 | DLX3 | 17q21.33 | distal-less homeobox 3 | protein-coding |
| 175 | AGA | 4q34.3 | aspartylglucosaminidase | protein-coding |
| 1756 | DMD | Xp21.2-p21.1 | dystrophin | protein-coding |
| 1786 | DNMT1 | 19p13.2 | DNA methyltransferase 1 | protein-coding |
| 1787 | TRDMT1 | 10p13 | tRNA aspartic acid methyltransferase 1 | protein-coding |
| 1788 | DNMT3A | 2p23.3 | DNA methyltransferase 3 alpha | protein-coding |
| 1789 | DNMT3B | 20q11.21 | DNA methyltransferase 3 beta | protein-coding |
| 1791 | DNTT | 10q24.1 | DNA nucleotidylexotransferase | protein-coding |
| 1794 | DOCK2 | 5q35.1 | dedicator of cytokinesis 2 | protein-coding |
| 1796 | DOK1 | 2p13.1 | docking protein 1 | protein-coding |
| 1801 | DPH1 | 17p13.3 | diphthamide biosynthesis 1 | protein-coding |
| 1803 | DPP4 | 2q24.2 | dipeptidyl peptidase 4 | protein-coding |
| 1804 | DPP6 | 7q36.2 | dipeptidyl peptidase like 6 | protein-coding |
| 182 | JAG1 | 20p12.2 | jagged 1 | protein-coding |
| 1820 | ARID3A | 19p13.3 | AT-rich interaction domain 3A | protein-coding |
| 183 | AGT | 1q42.2 | angiotensinogen | protein-coding |
| 1844 | DUSP2 | 2q11.2 | dual specificity phosphatase 2 | protein-coding |
| 1847 | DUSP5 | 10q25.2 | dual specificity phosphatase 5 | protein-coding |
| 1849 | DUSP7 | 3p21.2 | dual specificity phosphatase 7 | protein-coding |
| 185 | AGTR1 | 3q24 | angiotensin II receptor type 1 | protein-coding |
| 1859 | DYRK1A | 21q22.13 | dual specificity tyrosine phosphorylation regulated kinase 1A | protein-coding |
| 1869 | E2F1 | 20q11.22 | E2F transcription factor 1 | protein-coding |
| 1871 | E2F3 | 6p22.3 | E2F transcription factor 3 | protein-coding |
| 1877 | E4F1 | 16p13.3 | E4F transcription factor 1 | protein-coding |
| 1879 | EBF1 | 5q33.3 | early B-cell factor 1 | protein-coding |
| 1901 | S1PR1 | 1p21.2 | sphingosine-1-phosphate receptor 1 | protein-coding |
| 1902 | LPAR1 | 9q31.3 | lysophosphatidic acid receptor 1 | protein-coding |
| 1910 | EDNRB | 13q22.3 | endothelin receptor type B | protein-coding |
| 192343 | NEWENTRY | - | Record to support submission of GeneRIFs for a gene not in Gene (human; humans; man). | other |
| 1945 | EFNA4 | 1q21.3 | ephrin A4 | protein-coding |
| 1946 | EFNA5 | 5q21.3 | ephrin A5 | protein-coding |
| 1950 | EGF | 4q25 | epidermal growth factor | protein-coding |
| 1956 | EGFR | 7p11.2 | epidermal growth factor receptor | protein-coding |
| 1958 | EGR1 | 5q31.2 | early growth response 1 | protein-coding |
| 196 | AHR | 7p21.1 | aryl hydrocarbon receptor | protein-coding |
| 1969 | EPHA2 | 1p36.13 | EPH receptor A2 | protein-coding |
| 1977 | EIF4E | 4q23 | eukaryotic translation initiation factor 4E | protein-coding |
| 1978 | EIF4EBP1 | 8p11.23 | eukaryotic translation initiation factor 4E binding protein 1 | protein-coding |
| 1982 | EIF4G2 | 11p15.4 | eukaryotic translation initiation factor 4 gamma 2 | protein-coding |
| 1991 | ELANE | 19p13.3 | elastase, neutrophil expressed | protein-coding |
| 2000 | ELF4 | Xq26.1 | E74 like ETS transcription factor 4 | protein-coding |
| 2002 | ELK1 | Xp11.23 | ELK1, ETS transcription factor | protein-coding |
| 200424 | TET3 | 2p13.1 | tet methylcytosine dioxygenase 3 | protein-coding |
| 2012 | EMP1 | 12p13.1 | epithelial membrane protein 1 | protein-coding |
| 201294 | UNC13D | 17q25.1 | unc-13 homolog D | protein-coding |
| 2017 | CTTN | 11q13.3 | cortactin | protein-coding |
| 2020 | EN2 | 7q36.3 | engrailed homeobox 2 | protein-coding |
| 2022 | ENG | 9q34.11 | endoglin | protein-coding |
| 2026 | ENO2 | 12p13.31 | enolase 2 | protein-coding |
| 2033 | EP300 | 22q13.2 | E1A binding protein p300 | protein-coding |
| 2042 | EPHA3 | 3p11.1 | EPH receptor A3 | protein-coding |
| 2050 | EPHB4 | 7q22.1 | EPH receptor B4 | protein-coding |
| 2052 | EPHX1 | 1q42.12 | epoxide hydrolase 1 | protein-coding |
| 2056 | EPO | 7q22.1 | erythropoietin | protein-coding |
| 2057 | EPOR | 19p13.2 | erythropoietin receptor | protein-coding |
| 2059 | EPS8 | 12p12.3 | epidermal growth factor receptor pathway substrate 8 | protein-coding |
| 2060 | EPS15 | 1p32.3 | epidermal growth factor receptor pathway substrate 15 | protein-coding |
| 2064 | ERBB2 | 17q12 | erb-b2 receptor tyrosine kinase 2 | protein-coding |
| 2067 | ERCC1 | 19q13.32 | ERCC excision repair 1, endonuclease non-catalytic subunit | protein-coding |
| 2068 | ERCC2 | 19q13.32 | ERCC excision repair 2, TFIIH core complex helicase subunit | protein-coding |
| 207 | AKT1 | 14q32.33 | AKT serine/threonine kinase 1 | protein-coding |
| 2077 | ERF | 19q13.2 | ETS2 repressor factor | protein-coding |
| 2078 | ERG | 21q22.2 | ERG, ETS transcription factor | protein-coding |
| 208 | AKT2 | 19q13.2 | AKT serine/threonine kinase 2 | protein-coding |
| 2099 | ESR1 | 6q25.1-q25.2 | estrogen receptor 1 | protein-coding |
| 21 | ABCA3 | 16p13.3 | ATP binding cassette subfamily A member 3 | protein-coding |
| 2100 | ESR2 | 14q23.2-q23.3 | estrogen receptor 2 | protein-coding |
| 2113 | ETS1 | 11q24.3 | ETS proto-oncogene 1, transcription factor | protein-coding |
| 2114 | ETS2 | 21q22.2 | ETS proto-oncogene 2, transcription factor | protein-coding |
| 212 | ALAS2 | Xp11.21 | 5'-aminolevulinate synthase 2 | protein-coding |
| 2120 | ETV6 | 12p13.2 | ETS variant 6 | protein-coding |
| 2122 | MECOM | 3q26.2 | MDS1 and EVI1 complex locus | protein-coding |
| 213 | ALB | 4q13.3 | albumin | protein-coding |
| 2130 | EWSR1 | 22q12.2 | EWS RNA binding protein 1 | protein-coding |
| 214 | ALCAM | 3q13.11 | activated leukocyte cell adhesion molecule | protein-coding |
| 2146 | EZH2 | 7q36.1 | enhancer of zeste 2 polycomb repressive complex 2 subunit | protein-coding |
| 2149 | F2R | 5q13.3 | coagulation factor II thrombin receptor | protein-coding |
| 2152 | F3 | 1p21.3 | coagulation factor III, tissue factor | protein-coding |
| 2162 | F13A1 | 6p25.1 | coagulation factor XIII A chain | protein-coding |
| 2175 | FANCA | 16q24.3 | Fanconi anemia complementation group A | protein-coding |
| 2176 | FANCC | 9q22.32 | Fanconi anemia complementation group C | protein-coding |
| 2177 | FANCD2 | 3p25.3 | Fanconi anemia complementation group D2 | protein-coding |
| 2185 | PTK2B | 8p21.2 | protein tyrosine kinase 2 beta | protein-coding |
| 2189 | FANCG | 9p13.3 | Fanconi anemia complementation group G | protein-coding |
| 2195 | FAT1 | 4q35.2 | FAT atypical cadherin 1 | protein-coding |
| 2205 | FCER1A | 1q23.2 | Fc fragment of IgE receptor Ia | protein-coding |
| 2207 | FCER1G | 1q23.3 | Fc fragment of IgE receptor Ig | protein-coding |
| 2208 | FCER2 | 19p13.2 | Fc fragment of IgE receptor II | protein-coding |
| 221037 | JMJD1C | 10q21.3 | jumonji domain containing 1C | protein-coding |
| 2242 | FES | 15q26.1 | FES proto-oncogene, tyrosine kinase | protein-coding |
| 2247 | FGF2 | 4q28.1 | fibroblast growth factor 2 | protein-coding |
| 2249 | FGF4 | 11q13.3 | fibroblast growth factor 4 | protein-coding |
| 2252 | FGF7 | 15q21.2 | fibroblast growth factor 7 | protein-coding |
| 2260 | FGFR1 | 8p11.23 | fibroblast growth factor receptor 1 | protein-coding |
| 2261 | FGFR3 | 4p16.3 | fibroblast growth factor receptor 3 | protein-coding |
| 2268 | FGR | 1p35.3 | FGR proto-oncogene, Src family tyrosine kinase | protein-coding |
| 2272 | FHIT | 3p14.2 | fragile histidine triad | protein-coding |
| 2277 | VEGFD | Xp22.2 | vascular endothelial growth factor D | protein-coding |
| 22806 | IKZF3 | 17q12-q21.1 | IKAROS family zinc finger 3 | protein-coding |
| 22807 | IKZF2 | 2q34 | IKAROS family zinc finger 2 | protein-coding |
| 22809 | ATF5 | 19q13.33 | activating transcription factor 5 | protein-coding |
| 22877 | MLXIP | 12q24.31 | MLX interacting protein | protein-coding |
| 2289 | FKBP5 | 6p21.31 | FK506 binding protein 5 | protein-coding |
| 22914 | KLRK1 | 12p13.2 | killer cell lectin like receptor K1 | protein-coding |
| 22943 | DKK1 | 10q21.1 | dickkopf WNT signaling pathway inhibitor 1 | protein-coding |
| 22954 | TRIM32 | 9q33.1 | tripartite motif containing 32 | protein-coding |
| 22978 | NT5C2 | 10q24.32-q24.33 | 5'-nucleotidase, cytosolic II | protein-coding |
| 22985 | ACIN1 | 14q11.2 | apoptotic chromatin condensation inducer 1 | protein-coding |
| 23028 | KDM1A | 1p36.12 | lysine demethylase 1A | protein-coding |
| 23035 | PHLPP2 | 16q22.2 | PH domain and leucine rich repeat protein phosphatase 2 | protein-coding |
| 23049 | SMG1 | 16p12.3 | SMG1, nonsense mediated mRNA decay associated PI3K related kinase | protein-coding |
| 2305 | FOXM1 | 12p13.33 | forkhead box M1 | protein-coding |
| 2308 | FOXO1 | 13q14.11 | forkhead box O1 | protein-coding |
| 23085 | ERC1 | 12p13.33 | ELKS/RAB6-interacting/CAST family member 1 | protein-coding |
| 23087 | TRIM35 | 8p21.2 | tripartite motif containing 35 | protein-coding |
| 23089 | PEG10 | 7q21.3 | paternally expressed 10 | protein-coding |
| 2309 | FOXO3 | 6q21 | forkhead box O3 | protein-coding |
| 23090 | ZNF423 | 16q12.1 | zinc finger protein 423 | protein-coding |
| 23092 | ARHGAP26 | 5q31.3 | Rho GTPase activating protein 26 | protein-coding |
| 23095 | KIF1B | 1p36.22 | kinesin family member 1B | protein-coding |
| 2313 | FLI1 | 11q24.3 | Fli-1 proto-oncogene, ETS transcription factor | protein-coding |
| 23133 | PHF8 | Xp11.22 | PHD finger protein 8 | protein-coding |
| 23192 | ATG4B | 2q37.3 | autophagy related 4B cysteine peptidase | protein-coding |
| 2321 | FLT1 | 13q12.3 | fms related tyrosine kinase 1 | protein-coding |
| 2322 | FLT3 | 13q12.2 | fms related tyrosine kinase 3 | protein-coding |
| 23224 | SYNE2 | 14q23.2 | spectrin repeat containing nuclear envelope protein 2 | protein-coding |
| 2323 | FLT3LG | 19q13.33 | fms related tyrosine kinase 3 ligand | protein-coding |
| 23236 | PLCB1 | 20p12.3 | phospholipase C beta 1 | protein-coding |
| 23237 | ARC | 8q24.3 | activity regulated cytoskeleton associated protein | protein-coding |
| 23239 | PHLPP1 | 18q21.33 | PH domain and leucine rich repeat protein phosphatase 1 | protein-coding |
| 2324 | FLT4 | 5q35.3 | fms related tyrosine kinase 4 | protein-coding |
| 23250 | ATP11A | 13q34 | ATPase phospholipid transporting 11A | protein-coding |
| 23286 | WWC1 | 5q34 | WW and C2 domain containing 1 | protein-coding |
| 23308 | ICOSLG | 21q22.3 | inducible T-cell costimulator ligand | protein-coding |
| 2331 | FMOD | 1q32.1 | fibromodulin | protein-coding |
| 2335 | FN1 | 2q35 | fibronectin 1 | protein-coding |
| 23365 | ARHGEF12 | 11q23.3 | Rho guanine nucleotide exchange factor 12 | protein-coding |
| 23368 | PPP1R13B | 14q32.33 | protein phosphatase 1 regulatory subunit 13B | protein-coding |
| 23401 | FRAT2 | 10q24.1 | FRAT2, WNT signaling pathway regulator | protein-coding |
| 23405 | DICER1 | 14q32.13 | dicer 1, ribonuclease III | protein-coding |
| 23411 | SIRT1 | 10q21.3 | sirtuin 1 | protein-coding |
| 23435 | TARDBP | 1p36.22 | TAR DNA binding protein | protein-coding |
| 23451 | SF3B1 | 2q33.1 | splicing factor 3b subunit 1 | protein-coding |
| 23468 | CBX5 | 12q13.13 | chromobox 5 | protein-coding |
| 23476 | BRD4 | 19p13.12 | bromodomain containing 4 | protein-coding |
| 2348 | FOLR1 | 11q13.4 | folate receptor 1 | protein-coding |
| 23495 | TNFRSF13B | 17p11.2 | TNF receptor superfamily member 13B | protein-coding |
| 23512 | SUZ12 | 17q11.2 | SUZ12 polycomb repressive complex 2 subunit | protein-coding |
| 23532 | PRAME | 22q11.22 | preferentially expressed antigen in melanoma | protein-coding |
| 23547 | LILRA4 | 19q13.42 | leukocyte immunoglobulin like receptor A4 | protein-coding |
| 2355 | FOSL2 | 2p23.2 | FOS like 2, AP-1 transcription factor subunit | protein-coding |
| 2356 | FPGS | 9q34.11 | folylpolyglutamate synthase | protein-coding |
| 23566 | LPAR3 | 1p22.3 | lysophosphatidic acid receptor 3 | protein-coding |
| 23569 | PADI4 | 1p36.13 | peptidyl arginine deiminase 4 | protein-coding |
| 23581 | CASP14 | 19p13.12 | caspase 14 | protein-coding |
| 23586 | DDX58 | 9p21.1 | DExD/H-box helicase 58 | protein-coding |
| 23620 | NTSR2 | 2p25.1 | neurotensin receptor 2 | protein-coding |
| 23635 | SSBP2 | 5q14.1 | single stranded DNA binding protein 2 | protein-coding |
| 23640 | HSPBP1 | 19q13.42 | HSPA (Hsp70) binding protein 1 | protein-coding |
| 23641 | LDOC1 | Xq27.1 | leucine zipper down-regulated in cancer 1 | protein-coding |
| 23654 | PLXNB2 | 22q13.33 | plexin B2 | protein-coding |
| 23705 | CADM1 | 11q23.3 | cell adhesion molecule 1 | protein-coding |
| 23762 | OSBP2 | 22q12.2 | oxysterol binding protein 2 | protein-coding |
| 23786 | BCL2L13 | 22q11.21 | BCL2 like 13 | protein-coding |
| 238 | ALK | 2p23.2-p23.1 | anaplastic lymphoma receptor tyrosine kinase | protein-coding |
| 240 | ALOX5 | 10q11.21 | arachidonate 5-lipoxygenase | protein-coding |
| 246 | ALOX15 | 17p13.2 | arachidonate 15-lipoxygenase | protein-coding |
| 2475 | MTOR | 1p36.22 | mechanistic target of rapamycin | protein-coding |
| 2494 | NR5A2 | 1q32.1 | nuclear receptor subfamily 5 group A member 2 | protein-coding |
| 2495 | FTH1 | 11q12.3 | ferritin heavy chain 1 | protein-coding |
| 25 | ABL1 | 9q34.12 | ABL proto-oncogene 1, non-receptor tyrosine kinase | protein-coding |
| 2521 | FUS | 16p11.2 | FUS RNA binding protein | protein-coding |
| 2526 | FUT4 | 11q21 | fucosyltransferase 4 | protein-coding |
| 253260 | RICTOR | 5p13.1 | RPTOR independent companion of MTOR complex 2 | protein-coding |
| 2534 | FYN | 6q21 | FYN proto-oncogene, Src family tyrosine kinase | protein-coding |
| 2547 | XRCC6 | 22q13.2 | X-ray repair cross complementing 6 | protein-coding |
| 25800 | SLC39A6 | 18q12.2 | solute carrier family 39 member 6 | protein-coding |
| 25865 | PRKD2 | 19q13.32 | protein kinase D2 | protein-coding |
| 25875 | LETMD1 | 12q13.12 | LETM1 domain containing 1 | protein-coding |
| 25913 | POT1 | 7q31.33 | protection of telomeres 1 | protein-coding |
| 25939 | SAMHD1 | 20q11.23 | SAM and HD domain containing deoxynucleoside triphosphate triphosphohydrolase 1 | protein-coding |
| 2597 | GAPDH | 12p13.31 | glyceraldehyde-3-phosphate dehydrogenase | protein-coding |
| 26013 | L3MBTL1 | 20q13.12 | l(3)mbt-like 1 (Drosophila) | protein-coding |
| 26036 | ZNF451 | 6p12.1 | zinc finger protein 451 | protein-coding |
| 26038 | CHD5 | 1p36.31 | chromodomain helicase DNA binding protein 5 | protein-coding |
| 26040 | SETBP1 | 18q12.3 | SET binding protein 1 | protein-coding |
| 26053 | AUTS2 | 7q11.22 | autism susceptibility candidate 2 | protein-coding |
| 26122 | EPC2 | 2q23.1 | enhancer of polycomb homolog 2 | protein-coding |
| 26136 | TES | 7q31.2 | testin LIM domain protein | protein-coding |
| 26168 | SENP3 | 17p13.1 | SUMO1/sentrin/SMT3 specific peptidase 3 | protein-coding |
| 26191 | PTPN22 | 1p13.2 | protein tyrosine phosphatase, non-receptor type 22 | protein-coding |
| 2620 | GAS2 | 11p14.3 | growth arrest specific 2 | protein-coding |
| 2621 | GAS6 | 13q34 | growth arrest specific 6 | protein-coding |
| 2623 | GATA1 | Xp11.23 | GATA binding protein 1 | protein-coding |
| 2624 | GATA2 | 3q21.3 | GATA binding protein 2 | protein-coding |
| 2625 | GATA3 | 10p14 | GATA binding protein 3 | protein-coding |
| 26271 | FBXO5 | 6q25.2 | F-box protein 5 | protein-coding |
| 26354 | GNL3 | 3p21.1 | G protein nucleolar 3 | protein-coding |
| 2650 | GCNT1 | 9q21.13 | glucosaminyl (N-acetyl) transferase 1, core 2 | protein-coding |
| 26524 | LATS2 | 13q12.11 | large tumor suppressor kinase 2 | protein-coding |
| 267 | AMFR | 16q13 | autocrine motility factor receptor | protein-coding |
| 2671 | GFER | 16p13.3 | growth factor, augmenter of liver regeneration | protein-coding |
| 2672 | GFI1 | 1p22.1 | growth factor independent 1 transcriptional repressor | protein-coding |
| 2678 | GGT1 | 22q11.23 | gamma-glutamyltransferase 1 | protein-coding |
| 2683 | B4GALT1 | 9p21.1 | beta-1,4-galactosyltransferase 1 | protein-coding |
| 2694 | GIF | 11q12.1 | gastric intrinsic factor | protein-coding |
| 2697 | GJA1 | 6q22.31 | gap junction protein alpha 1 | protein-coding |
| 26973 | CHORDC1 | 11q14.3 | cysteine and histidine rich domain containing 1 | protein-coding |
| 27 | ABL2 | 1q25.2 | ABL proto-oncogene 2, non-receptor tyrosine kinase | protein-coding |
| 27000 | DNAJC2 | 7q22.1 | DnaJ heat shock protein family (Hsp40) member C2 | protein-coding |
| 27035 | NOX1 | Xq22.1 | NADPH oxidase 1 | protein-coding |
| 27036 | SIGLEC7 | 19q13.41 | sialic acid binding Ig like lectin 7 | protein-coding |
| 27086 | FOXP1 | 3p13 | forkhead box P1 | protein-coding |
| 27087 | B3GAT1 | 11q25 | beta-1,3-glucuronyltransferase 1 | protein-coding |
| 27113 | BBC3 | 19q13.32 | BCL2 binding component 3 | protein-coding |
| 27115 | PDE7B | 6q23.3 | phosphodiesterase 7B | protein-coding |
| 27125 | AFF4 | 5q31.1 | AF4/FMR2 family member 4 | protein-coding |
| 27161 | AGO2 | 8q24.3 | argonaute 2, RISC catalytic component | protein-coding |
| 27180 | SIGLEC9 | 19q13.41 | sialic acid binding Ig like lectin 9 | protein-coding |
| 27181 | SIGLEC8 | 19q13.41 | sialic acid binding Ig like lectin 8 | protein-coding |
| 2735 | GLI1 | 12q13.3 | GLI family zinc finger 1 | protein-coding |
| 2739 | GLO1 | 6p21.2 | glyoxalase I | protein-coding |
| 27429 | HTRA2 | 2p13.1 | HtrA serine peptidase 2 | protein-coding |
| 2744 | GLS | 2q32.2 | glutaminase | protein-coding |
| 2768 | GNA12 | 7p22.3-p22.2 | G protein subunit alpha 12 | protein-coding |
| 2778 | GNAS | 20q13.32 | GNAS complex locus | protein-coding |
| 2822 | GPLD1 | 6p22.3 | glycosylphosphatidylinositol specific phospholipase D1 | protein-coding |
| 2823 | GPM6A | 4q34.2 | glycoprotein M6A | protein-coding |
| 28234 | SLCO1B3 | 12p12.2 | solute carrier organic anion transporter family member 1B3 | protein-coding |
| 2824 | GPM6B | Xp22.2 | glycoprotein M6B | protein-coding |
| 2826 | CCR10 | 17q21.2 | C-C motif chemokine receptor 10 | protein-coding |
| 283 | ANG | 14q11.2 | angiogenin | protein-coding |
| 283120 | H19 | 11p15.5 | H19, imprinted maternally expressed transcript (non-protein coding) | ncRNA |
| 2833 | CXCR3 | Xq13.1 | C-X-C motif chemokine receptor 3 | protein-coding |
| 283455 | KSR2 | 12q24.22-q24.23 | kinase suppressor of ras 2 | protein-coding |
| 283518 | KCNRG | 13q14.2 | potassium channel regulator | protein-coding |
| 283871 | PGP | 16p13.3 | phosphoglycolate phosphatase | protein-coding |
| 28392 | IGHV4-59 | 14q32.33 | immunoglobulin heavy variable 4-59 | other |
| 28396 | IGHV4-31 | 14q32.33 | immunoglobulin heavy variable 4-31 | other |
| 284 | ANGPT1 | 8q23.1 | angiopoietin 1 | protein-coding |
| 28444 | IGHV3-21 | 14q32.33 | immunoglobulin heavy variable 3-21 | other |
| 28452 | IGHV3-7 | 14q32.33 | immunoglobulin heavy variable 3-7 | other |
| 2846 | LPAR4 | Xq21.1 | lysophosphatidic acid receptor 4 | protein-coding |
| 285 | ANGPT2 | 8p23.1 | angiopoietin 2 | protein-coding |
| 28514 | DLL1 | 6q27 | delta like canonical Notch ligand 1 | protein-coding |
| 285527 | FRYL | 4p11 | FRY like transcription coactivator | protein-coding |
| 286053 | NSMCE2 | 8q24.13 | NSE2/MMS21 homolog, SMC5-SMC6 complex SUMO ligase | protein-coding |
| 286530 | P2RY8 | Xp22.33 and Yp11.2 | purinergic receptor P2Y8 | protein-coding |
| 2872 | MKNK2 | 19p13.3 | MAP kinase interacting serine/threonine kinase 2 | protein-coding |
| 2878 | GPX3 | 5q33.1 | glutathione peroxidase 3 | protein-coding |
| 2885 | GRB2 | 17q25.1 | growth factor receptor bound protein 2 | protein-coding |
| 2886 | GRB7 | 17q12 | growth factor receptor bound protein 7 | protein-coding |
| 2887 | GRB10 | 7p12.1 | growth factor receptor bound protein 10 | protein-coding |
| 2889 | RAPGEF1 | 9q34.13 | Rap guanine nucleotide exchange factor 1 | protein-coding |
| 2892 | GRIA3 | Xq25 | glutamate ionotropic receptor AMPA type subunit 3 | protein-coding |
| 28951 | TRIB2 | 2p24.3 | tribbles pseudokinase 2 | protein-coding |
| 2896 | GRN | 17q21.31 | granulin precursor | protein-coding |
| 28992 | MACROD1 | 11q13.1 | MACRO domain containing 1 | protein-coding |
| 28996 | HIPK2 | 7q34 | homeodomain interacting protein kinase 2 | protein-coding |
| 290 | ANPEP | 15q26.1 | alanyl aminopeptidase, membrane | protein-coding |
| 29072 | SETD2 | 3p21.31 | SET domain containing 2 | protein-coding |
| 2908 | NR3C1 | 5q31.3 | nuclear receptor subfamily 3 group C member 1 | protein-coding |
| 291 | SLC25A4 | 4q35.1 | solute carrier family 25 member 4 | protein-coding |
| 29102 | DROSHA | 5p13.3 | drosha ribonuclease III | protein-coding |
| 29108 | PYCARD | 16p11.2 | PYD and CARD domain containing | protein-coding |
| 29126 | CD274 | 9p24.1 | CD274 molecule | protein-coding |
| 2919 | CXCL1 | 4q13.3 | C-X-C motif chemokine ligand 1 | protein-coding |
| 2931 | GSK3A | 19q13.2 | glycogen synthase kinase 3 alpha | protein-coding |
| 2932 | GSK3B | 3q13.33 | glycogen synthase kinase 3 beta | protein-coding |
| 2936 | GSR | 8p12 | glutathione-disulfide reductase | protein-coding |
| 2938 | GSTA1 | 6p12.2 | glutathione S-transferase alpha 1 | protein-coding |
| 2941 | GSTA4 | 6p12.2 | glutathione S-transferase alpha 4 | protein-coding |
| 2944 | GSTM1 | 1p13.3 | glutathione S-transferase mu 1 | protein-coding |
| 2950 | GSTP1 | 11q13.2 | glutathione S-transferase pi 1 | protein-coding |
| 2952 | GSTT1 | 22q11.23 | glutathione S-transferase theta 1 | protein-coding |
| 29760 | BLNK | 10q24.1 | B-cell linker | protein-coding |
| 29843 | SENP1 | 12q13.11 | SUMO1/sentrin specific peptidase 1 | protein-coding |
| 29952 | DPP7 | 9q34.3 | dipeptidyl peptidase 7 | protein-coding |
| 29959 | NRBP1 | 2p23.3 | nuclear receptor binding protein 1 | protein-coding |
| 2997 | GYS1 | 19q13.33 | glycogen synthase 1 | protein-coding |
| 30009 | TBX21 | 17q21.32 | T-box 21 | protein-coding |
| 30012 | TLX3 | 5q35.1 | T-cell leukemia homeobox 3 | protein-coding |
| 3002 | GZMB | 14q12 | granzyme B | protein-coding |
| 301 | ANXA1 | 9q21.13 | annexin A1 | protein-coding |
| 3014 | H2AFX | 11q23.3 | H2A histone family member X | protein-coding |
| 302 | ANXA2 | 15q22.2 | annexin A2 | protein-coding |
| 3020 | H3F3A | 1q42.12 | H3 histone family member 3A | protein-coding |
| 3024 | HIST1H1A | 6p22.2 | histone cluster 1 H1 family member a | protein-coding |
| 3036 | HAS1 | 19q13.41 | hyaluronan synthase 1 | protein-coding |
| 3054 | HCFC1 | Xq28 | host cell factor C1 | protein-coding |
| 3055 | HCK | 20q11.21 | HCK proto-oncogene, Src family tyrosine kinase | protein-coding |
| 3059 | HCLS1 | 3q13.33 | hematopoietic cell-specific Lyn substrate 1 | protein-coding |
| 3064 | HTT | 4p16.3 | huntingtin | protein-coding |
| 3065 | HDAC1 | 1p35.2-p35.1 | histone deacetylase 1 | protein-coding |
| 3067 | HDC | 15q21.2 | histidine decarboxylase | protein-coding |
| 3070 | HELLS | 10q23.33 | helicase, lymphoid-specific | protein-coding |
| 3077 | HFE | 6p22.2 | hemochromatosis | protein-coding |
| 30818 | KCNIP3 | 2q11.1 | potassium voltage-gated channel interacting protein 3 | protein-coding |
| 3082 | HGF | 7q21.11 | hepatocyte growth factor | protein-coding |
| 30849 | PIK3R4 | 3q22.1 | phosphoinositide-3-kinase regulatory subunit 4 | protein-coding |
| 3087 | HHEX | 10q23.33 | hematopoietically expressed homeobox | protein-coding |
| 3091 | HIF1A | 14q23.2 | hypoxia inducible factor 1 alpha subunit | protein-coding |
| 3101 | HK3 | 5q35.2 | hexokinase 3 | protein-coding |
| 3105 | HLA-A | 6p22.1 | major histocompatibility complex, class I, A | protein-coding |
| 3106 | HLA-B | 6p21.33 | major histocompatibility complex, class I, B | protein-coding |
| 3110 | MNX1 | 7q36.3 | motor neuron and pancreas homeobox 1 | protein-coding |
| 3111 | HLA-DOA | 6p21.32 | major histocompatibility complex, class II, DO alpha | protein-coding |
| 3112 | HLA-DOB | 6p21.32 | major histocompatibility complex, class II, DO beta | protein-coding |
| 3113 | HLA-DPA1 | 6p21.32 | major histocompatibility complex, class II, DP alpha 1 | protein-coding |
| 3115 | HLA-DPB1 | 6p21.32 | major histocompatibility complex, class II, DP beta 1 | protein-coding |
| 3117 | HLA-DQA1 | 6p21.32 | major histocompatibility complex, class II, DQ alpha 1 | protein-coding |
| 3119 | HLA-DQB1 | 6p21.32 | major histocompatibility complex, class II, DQ beta 1 | protein-coding |
| 3122 | HLA-DRA | 6p21.32 | major histocompatibility complex, class II, DR alpha | protein-coding |
| 3123 | HLA-DRB1 | 6p21.32 | major histocompatibility complex, class II, DR beta 1 | protein-coding |
| 3126 | HLA-DRB4 | 6p21.3 | major histocompatibility complex, class II, DR beta 4 | protein-coding |
| 3131 | HLF | 17q22 | HLF, PAR bZIP transcription factor | protein-coding |
| 3133 | HLA-E | 6p22.1 | major histocompatibility complex, class I, E | protein-coding |
| 3135 | HLA-G | 6p22.1 | major histocompatibility complex, class I, G | protein-coding |
| 3140 | MR1 | 1q25.3 | major histocompatibility complex, class I-related | protein-coding |
| 3142 | HLX | 1q41 | H2.0 like homeobox | protein-coding |
| 3146 | HMGB1 | 13q12.3 | high mobility group box 1 | protein-coding |
| 3159 | HMGA1 | 6p21.31 | high mobility group AT-hook 1 | protein-coding |
| 3161 | HMMR | 5q34 | hyaluronan mediated motility receptor | protein-coding |
| 3162 | HMOX1 | 22q12.3 | heme oxygenase 1 | protein-coding |
| 3164 | NR4A1 | 12q13.13 | nuclear receptor subfamily 4 group A member 1 | protein-coding |
| 317 | APAF1 | 12q23.1 | apoptotic peptidase activating factor 1 | protein-coding |
| 3177 | SLC29A2 | 11q13.2 | solute carrier family 29 member 2 | protein-coding |
| 3187 | HNRNPH1 | 5q35.3 | heterogeneous nuclear ribonucleoprotein H1 (H) | protein-coding |
| 3190 | HNRNPK | 9q21.32 | heterogeneous nuclear ribonucleoprotein K | protein-coding |
| 3195 | TLX1 | 10q24.31 | T-cell leukemia homeobox 1 | protein-coding |
| 3197 | HOXA@ | 7p15.2 | homeobox A cluster | other |
| 3201 | HOXA4 | 7p15.2 | homeobox A4 | protein-coding |
| 3202 | HOXA5 | 7p15.2 | homeobox A5 | protein-coding |
| 3203 | HOXA6 | 7p15.2 | homeobox A6 | protein-coding |
| 3204 | HOXA7 | 7p15.2 | homeobox A7 | protein-coding |
| 3205 | HOXA9 | 7p15.2 | homeobox A9 | protein-coding |
| 3206 | HOXA10 | 7p15.2 | homeobox A10 | protein-coding |
| 3207 | HOXA11 | 7p15.2 | homeobox A11 | protein-coding |
| 3209 | HOXA13 | 7p15.2 | homeobox A13 | protein-coding |
| 3214 | HOXB4 | 17q21.32 | homeobox B4 | protein-coding |
| 3216 | HOXB6 | 17q21.32 | homeobox B6 | protein-coding |
| 3232 | HOXD3 | 2q31.1 | homeobox D3 | protein-coding |
| 3236 | HOXD10 | 2q31.1 | homeobox D10 | protein-coding |
| 3237 | HOXD11 | 2q31.1 | homeobox D11 | protein-coding |
| 3239 | HOXD13 | 2q31.1 | homeobox D13 | protein-coding |
| 324 | APC | 5q22.2 | APC, WNT signaling pathway regulator | protein-coding |
| 3251 | HPRT1 | Xq26.2-q26.3 | hypoxanthine phosphoribosyltransferase 1 | protein-coding |
| 3265 | HRAS | 11p15.5 | HRas proto-oncogene, GTPase | protein-coding |
| 3276 | PRMT1 | 19q13.33 | protein arginine methyltransferase 1 | protein-coding |
| 328 | APEX1 | 14q11.2 | apurinic/apyrimidinic endodeoxyribonuclease 1 | protein-coding |
| 3280 | HES1 | 3q29 | hes family bHLH transcription factor 1 | protein-coding |
| 329 | BIRC2 | 11q22.2 | baculoviral IAP repeat containing 2 | protein-coding |
| 3291 | HSD11B2 | 16q22.1 | hydroxysteroid 11-beta dehydrogenase 2 | protein-coding |
| 3292 | HSD17B1 | 17q21.2 | hydroxysteroid 17-beta dehydrogenase 1 | protein-coding |
| 3297 | HSF1 | 8q24.3 | heat shock transcription factor 1 | protein-coding |
| 3298 | HSF2 | 6q22.31 | heat shock transcription factor 2 | protein-coding |
| 330 | BIRC3 | 11q22.2 | baculoviral IAP repeat containing 3 | protein-coding |
| 3303 | HSPA1A | 6p21.33 | heat shock protein family A (Hsp70) member 1A | protein-coding |
| 3304 | HSPA1B | 6p21.33 | heat shock protein family A (Hsp70) member 1B | protein-coding |
| 3305 | HSPA1L | 6p21.33 | heat shock protein family A (Hsp70) member 1 like | protein-coding |
| 3308 | HSPA4 | 5q31.1 | heat shock protein family A (Hsp70) member 4 | protein-coding |
| 3309 | HSPA5 | 9q33.3 | heat shock protein family A (Hsp70) member 5 | protein-coding |
| 331 | XIAP | Xq25 | X-linked inhibitor of apoptosis | protein-coding |
| 3315 | HSPB1 | 7q11.23 | heat shock protein family B (small) member 1 | protein-coding |
| 332 | BIRC5 | 17q25.3 | baculoviral IAP repeat containing 5 | protein-coding |
| 3320 | HSP90AA1 | 14q32.31 | heat shock protein 90 alpha family class A member 1 | protein-coding |
| 3324 | HSP90AA2P | 11p14.1 | heat shock protein 90 alpha family class A member 2, pseudogene | pseudo |
| 3326 | HSP90AB1 | 6p21.1 | heat shock protein 90 alpha family class B member 1 | protein-coding |
| 3329 | HSPD1 | 2q33.1 | heat shock protein family D (Hsp60) member 1 | protein-coding |
| 3337 | DNAJB1 | 19p13.12 | DnaJ heat shock protein family (Hsp40) member B1 | protein-coding |
| 3383 | ICAM1 | 19p13.2 | intercellular adhesion molecule 1 | protein-coding |
| 338436 | BLACE | 7q36.3 | B-cell acute lymphoblastic leukemia expressed | ncRNA |
| 338567 | KCNK18 | 10q25.3 | potassium two pore domain channel subfamily K member 18 | protein-coding |
| 3394 | IRF8 | 16q24.1 | interferon regulatory factor 8 | protein-coding |
| 3397 | ID1 | 20q11.21 | inhibitor of DNA binding 1, HLH protein | protein-coding |
| 3398 | ID2 | 2p25.1 | inhibitor of DNA binding 2, HLH protein | protein-coding |
| 3399 | ID3 | 1p36.12 | inhibitor of DNA binding 3, HLH protein | protein-coding |
| 3400 | ID4 | 6p22.3 | inhibitor of DNA binding 4, HLH protein | protein-coding |
| 340273 | ABCB5 | 7p21.1 | ATP binding cassette subfamily B member 5 | protein-coding |
| 3417 | IDH1 | 2q34 | isocitrate dehydrogenase (NADP(+)) 1, cytosolic | protein-coding |
| 3418 | IDH2 | 15q26.1 | isocitrate dehydrogenase (NADP(+)) 2, mitochondrial | protein-coding |
| 343641 | TGM6 | 20p13 | transglutaminase 6 | protein-coding |
| 3439 | IFNA1 | 9p21.3 | interferon alpha 1 | protein-coding |
| 3454 | IFNAR1 | 21q22.11 | interferon alpha and beta receptor subunit 1 | protein-coding |
| 3458 | IFNG | 12q15 | interferon gamma | protein-coding |
| 3459 | IFNGR1 | 6q23.3 | interferon gamma receptor 1 | protein-coding |
| 3478 | IGES | 5q31.1 | immunoglobulin E concentration, serum | unknown |
| 3479 | IGF1 | 12q23.2 | insulin like growth factor 1 | protein-coding |
| 348 | APOE | 19q13.32 | apolipoprotein E | protein-coding |
| 3480 | IGF1R | 15q26.3 | insulin like growth factor 1 receptor | protein-coding |
| 3481 | IGF2 | 11p15.5 | insulin like growth factor 2 | protein-coding |
| 3485 | IGFBP2 | 2q35 | insulin like growth factor binding protein 2 | protein-coding |
| 3490 | IGFBP7 | 4q12 | insulin like growth factor binding protein 7 | protein-coding |
| 3492 | IGH | 14q32.33 | immunoglobulin heavy locus | protein-coding |
| 3495 | IGHD | 14q32.33 | immunoglobulin heavy constant delta | other |
| 3507 | IGHM | 14q32.33 | immunoglobulin heavy constant mu | other |
| 351 | APP | 21q21.3 | amyloid beta precursor protein | protein-coding |
| 3535 | IGL | 22q11.22 | immunoglobulin lambda locus | protein-coding |
| 355 | FAS | 10q23.31 | Fas cell surface death receptor | protein-coding |
| 3552 | IL1A | 2q14.1 | interleukin 1 alpha | protein-coding |
| 3553 | IL1B | 2q14.1 | interleukin 1 beta | protein-coding |
| 3556 | IL1RAP | 3q28 | interleukin 1 receptor accessory protein | protein-coding |
| 3557 | IL1RN | 2q14.1 | interleukin 1 receptor antagonist | protein-coding |
| 3558 | IL2 | 4q27 | interleukin 2 | protein-coding |
| 3559 | IL2RA | 10p15.1 | interleukin 2 receptor subunit alpha | protein-coding |
| 356 | FASLG | 1q24.3 | Fas ligand | protein-coding |
| 3560 | IL2RB | 22q12.3 | interleukin 2 receptor subunit beta | protein-coding |
| 3561 | IL2RG | Xq13.1 | interleukin 2 receptor subunit gamma | protein-coding |
| 3563 | IL3RA | Xp22.33 and Yp11.2 | interleukin 3 receptor subunit alpha | protein-coding |
| 3565 | IL4 | 5q31.1 | interleukin 4 | protein-coding |
| 3566 | IL4R | 16p12.1 | interleukin 4 receptor | protein-coding |
| 3567 | IL5 | 5q31.1 | interleukin 5 | protein-coding |
| 3569 | IL6 | 7p15.3 | interleukin 6 | protein-coding |
| 3572 | IL6ST | 5q11.2 | interleukin 6 signal transducer | protein-coding |
| 3574 | IL7 | 8q21.13 | interleukin 7 | protein-coding |
| 3575 | IL7R | 5p13.2 | interleukin 7 receptor | protein-coding |
| 3576 | CXCL8 | 4q13.3 | C-X-C motif chemokine ligand 8 | protein-coding |
| 3577 | CXCR1 | 2q35 | C-X-C motif chemokine receptor 1 | protein-coding |
| 3578 | IL9 | 5q31.1 | interleukin 9 | protein-coding |
| 3579 | CXCR2 | 2q35 | C-X-C motif chemokine receptor 2 | protein-coding |
| 3586 | IL10 | 1q32.1 | interleukin 10 | protein-coding |
| 3589 | IL11 | 19q13.42 | interleukin 11 | protein-coding |
| 3592 | IL12A | 3q25.33 | interleukin 12A | protein-coding |
| 3600 | IL15 | 4q31.21 | interleukin 15 | protein-coding |
| 3601 | IL15RA | 10p15.1 | interleukin 15 receptor subunit alpha | protein-coding |
| 3604 | TNFRSF9 | 1p36.23 | TNF receptor superfamily member 9 | protein-coding |
| 3605 | IL17A | 6p12.2 | interleukin 17A | protein-coding |
| 3606 | IL18 | 11q23.1 | interleukin 18 | protein-coding |
| 3613 | IMPA2 | 18p11.21 | inositol monophosphatase 2 | protein-coding |
| 362 | AQP5 | 12q13.12 | aquaporin 5 | protein-coding |
| 3620 | IDO1 | 8p11.21 | indoleamine 2,3-dioxygenase 1 | protein-coding |
| 3627 | CXCL10 | 4q21.1 | C-X-C motif chemokine ligand 10 | protein-coding |
| 3635 | INPP5D | 2q37.1 | inositol polyphosphate-5-phosphatase D | protein-coding |
| 3636 | INPPL1 | 11q13.4 | inositol polyphosphate phosphatase like 1 | protein-coding |
| 3643 | INSR | 19p13.2 | insulin receptor | protein-coding |
| 366 | AQP9 | 15q21.3 | aquaporin 9 | protein-coding |
| 3661 | IRF3 | 19q13.33 | interferon regulatory factor 3 | protein-coding |
| 3662 | IRF4 | 6p25.3 | interferon regulatory factor 4 | protein-coding |
| 3663 | IRF5 | 7q32.1 | interferon regulatory factor 5 | protein-coding |
| 367 | AR | Xq12 | androgen receptor | protein-coding |
| 3673 | ITGA2 | 5q11.2 | integrin subunit alpha 2 | protein-coding |
| 3674 | ITGA2B | 17q21.31 | integrin subunit alpha 2b | protein-coding |
| 3676 | ITGA4 | 2q31.3 | integrin subunit alpha 4 | protein-coding |
| 3683 | ITGAL | 16p11.2 | integrin subunit alpha L | protein-coding |
| 3684 | ITGAM | 16p11.2 | integrin subunit alpha M | protein-coding |
| 3687 | ITGAX | 16p11.2 | integrin subunit alpha X | protein-coding |
| 3688 | ITGB1 | 10p11.22 | integrin subunit beta 1 | protein-coding |
| 3689 | ITGB2 | 21q22.3 | integrin subunit beta 2 | protein-coding |
| 3690 | ITGB3 | 17q21.32 | integrin subunit beta 3 | protein-coding |
| 3695 | ITGB7 | 12q13.13 | integrin subunit beta 7 | protein-coding |
| 3704 | ITPA | 20p13 | inosine triphosphatase | protein-coding |
| 3714 | JAG2 | 14q32.33 | jagged 2 | protein-coding |
| 3716 | JAK1 | 1p31.3 | Janus kinase 1 | protein-coding |
| 3717 | JAK2 | 9p24.1 | Janus kinase 2 | protein-coding |
| 3718 | JAK3 | 19p13.11 | Janus kinase 3 | protein-coding |
| 3725 | JUN | 1p32.1 | Jun proto-oncogene, AP-1 transcription factor subunit | protein-coding |
| 3726 | JUNB | 19p13.13 | JunB proto-oncogene, AP-1 transcription factor subunit | protein-coding |
| 3727 | JUND | 19p13.11 | JunD proto-oncogene, AP-1 transcription factor subunit | protein-coding |
| 3728 | JUP | 17q21.2 | junction plakoglobin | protein-coding |
| 373156 | GSTK1 | 7q34 | glutathione S-transferase kappa 1 | protein-coding |
| 3732 | CD82 | 11p11.2 | CD82 molecule | protein-coding |
| 3738 | KCNA3 | 1p13.3 | potassium voltage-gated channel subfamily A member 3 | protein-coding |
| 375 | ARF1 | 1q42.13 | ADP ribosylation factor 1 | protein-coding |
| 3756 | KCNH1 | 1q32.2 | potassium voltage-gated channel subfamily H member 1 | protein-coding |
| 3757 | KCNH2 | 7q36.1 | potassium voltage-gated channel subfamily H member 2 | protein-coding |
| 3791 | KDR | 4q12 | kinase insert domain receptor | protein-coding |
| 3802 | KIR2DL1 | 19q13.42 | killer cell immunoglobulin like receptor, two Ig domains and long cytoplasmic tail 1 | protein-coding |
| 3803 | KIR2DL2 | 19q13.4 | killer cell immunoglobulin like receptor, two Ig domains and long cytoplasmic tail 2 | protein-coding |
| 3806 | KIR2DS1 | 19q13.4 | killer cell immunoglobulin like receptor, two Ig domains and short cytoplasmic tail 1 | protein-coding |
| 3808 | KIR2DS3 | 19q13.4 | killer cell immunoglobulin like receptor, two Ig domains and short cytoplasmic tail 3 | protein-coding |
| 3809 | KIR2DS4 | 19q13.42 | killer cell immunoglobulin like receptor, two Ig domains and short cytoplasmic tail 4 | protein-coding |
| 3810 | KIR2DS5 | 19q13.4 | killer cell immunoglobulin like receptor, two Ig domains and short cytoplasmic tail 5 | protein-coding |
| 3811 | KIR3DL1 | 19q13.42 | killer cell immunoglobulin like receptor, three Ig domains and long cytoplasmic tail 1 | protein-coding |
| 3812 | KIR3DL2 | 19q13.42 | killer cell immunoglobulin like receptor, three Ig domains and long cytoplasmic tail 2 | protein-coding |
| 3815 | KIT | 4q12 | KIT proto-oncogene receptor tyrosine kinase | protein-coding |
| 3821 | KLRC1 | 12p13.2 | killer cell lectin like receptor C1 | protein-coding |
| 3824 | KLRD1 | 12p13.2 | killer cell lectin like receptor D1 | protein-coding |
| 3845 | KRAS | 12p12.1 | KRAS proto-oncogene, GTPase | protein-coding |
| 387 | RHOA | 3p21.31 | ras homolog family member A | protein-coding |
| 387893 | KMT5A | 12q24.31 | lysine methyltransferase 5A | protein-coding |
| 388324 | INCA1 | 17p13.2 | inhibitor of CDK, cyclin A1 interacting protein 1 | protein-coding |
| 388585 | HES5 | 1p36.32 | hes family bHLH transcription factor 5 | protein-coding |
| 388815 | MIR99AHG | 21q21.1 | mir-99a-let-7c cluster host gene | ncRNA |
| 3899 | AFF3 | 2q11.2 | AF4/FMR2 family member 3 | protein-coding |
| 3903 | LAIR1 | 19q13.42 | leukocyte associated immunoglobulin like receptor 1 | protein-coding |
| 3916 | LAMP1 | 13q34 | lysosomal associated membrane protein 1 | protein-coding |
| 3921 | RPSA | 3p22.1 | ribosomal protein SA | protein-coding |
| 3925 | STMN1 | 1p36.11 | stathmin 1 | protein-coding |
| 3932 | LCK | 1p35.2 | LCK proto-oncogene, Src family tyrosine kinase | protein-coding |
| 3934 | LCN2 | 9q34.11 | lipocalin 2 | protein-coding |
| 3939 | LDHA | 11p15.1 | lactate dehydrogenase A | protein-coding |
| 3952 | LEP | 7q32.1 | leptin | protein-coding |
| 3953 | LEPR | 1p31.3 | leptin receptor | protein-coding |
| 3958 | LGALS3 | 14q22.3 | galectin 3 | protein-coding |
| 3965 | LGALS9 | 17q11.2 | galectin 9 | protein-coding |
| 3976 | LIF | 22q12.2 | leukemia inhibitory factor | protein-coding |
| 3977 | LIFR | 5p13.1 | leukemia inhibitory factor receptor alpha | protein-coding |
| 3980 | LIG3 | 17q12 | DNA ligase 3 | protein-coding |
| 3981 | LIG4 | 13q33.3 | DNA ligase 4 | protein-coding |
| 3987 | LIMS1 | 2q12.3 | LIM zinc finger domain containing 1 | protein-coding |
| 399 | RHOH | 4p14 | ras homolog family member H | protein-coding |
| 3996 | LLGL1 | 17p11.2 | LLGL1, scribble cell polarity complex component | protein-coding |
| 399959 | MIR100HG | 11q24.1 | mir-100-let-7a-2 cluster host gene | ncRNA |
| 4004 | LMO1 | 11p15.4 | LIM domain only 1 | protein-coding |
| 4005 | LMO2 | 11p13 | LIM domain only 2 | protein-coding |
| 4023 | LPL | 8p21.3 | lipoprotein lipase | protein-coding |
| 4035 | LRP1 | 12q13.3 | LDL receptor related protein 1 | protein-coding |
| 4040 | LRP6 | 12p13.2 | LDL receptor related protein 6 | protein-coding |
| 4041 | LRP5 | 11q13.2 | LDL receptor related protein 5 | protein-coding |
| 4049 | LTA | 6p21.33 | lymphotoxin alpha | protein-coding |
| 405 | ARNT | 1q21.3 | aryl hydrocarbon receptor nuclear translocator | protein-coding |
| 4056 | LTC4S | 5q35.3 | leukotriene C4 synthase | protein-coding |
| 4057 | LTF | 3p21.31 | lactotransferrin | protein-coding |
| 4065 | LY75 | 2q24.2 | lymphocyte antigen 75 | protein-coding |
| 4066 | LYL1 | 19p13.13 | LYL1, basic helix-loop-helix family member | protein-coding |
| 4067 | LYN | 8q12.1 | LYN proto-oncogene, Src family tyrosine kinase | protein-coding |
| 406883 | MIRLET7A3 | 22q13.31 | microRNA let-7a-3 | ncRNA |
| 406884 | MIRLET7B | 22q13.31 | microRNA let-7b | ncRNA |
| 406885 | MIRLET7C | 21q21.1 | microRNA let-7c | ncRNA |
| 406892 | MIR100 | 11q24.1 | microRNA 100 | ncRNA |
| 406893 | MIR101-1 | 1p31.3 | microRNA 101-1 | ncRNA |
| 4069 | LYZ | 12q15 | lysozyme | protein-coding |
| 406900 | MIR106B | 7q22.1 | microRNA 106b | ncRNA |
| 406902 | MIR10A | 17q21.32 | microRNA 10a | ncRNA |
| 406905 | MIR1-2 | 18q11.2 | microRNA 1-2 | ncRNA |
| 406907 | MIR124-1 | 8p23.1 | microRNA 124-1 | ncRNA |
| 406910 | MIR125A | 19q13.41 | microRNA 125a | ncRNA |
| 406911 | MIR125B1 | 11q24.1 | microRNA 125b-1 | ncRNA |
| 406912 | MIR125B2 | 21q21.1 | microRNA 125b-2 | ncRNA |
| 406913 | MIR126 | 9q34.3 | microRNA 126 | ncRNA |
| 406916 | MIR128-2 | 3p22.3 | microRNA 128-2 | ncRNA |
| 406921 | MIR132 | 17p13.3 | microRNA 132 | ncRNA |
| 406929 | MIR138-1 | 3p21.32 | microRNA 138-1 | ncRNA |
| 406934 | MIR142 | 17q22 | microRNA 142 | ncRNA |
| 406935 | MIR143 | 5q32 | microRNA 143 | ncRNA |
| 406937 | MIR145 | 5q32 | microRNA 145 | ncRNA |
| 406938 | MIR146A | 5q33.3 | microRNA 146a | ncRNA |
| 406942 | MIR150 | 19q13.33 | microRNA 150 | ncRNA |
| 406947 | MIR155 | 21q21.3 | microRNA 155 | ncRNA |
| 406948 | MIR15A | 13q14.2 | microRNA 15a | ncRNA |
| 406950 | MIR16-1 | 13q14.2 | microRNA 16-1 | ncRNA |
| 406952 | MIR17 | 13q31.3 | microRNA 17 | ncRNA |
| 406955 | MIR181B1 | 1q32.1 | microRNA 181b-1 | ncRNA |
| 406956 | MIR181B2 | 9q33.3 | microRNA 181b-2 | ncRNA |
| 406959 | MIR183 | 7q32.2 | microRNA 183 | ncRNA |
| 406973 | MIR196A2 | 12q13.13 | microRNA 196a-2 | ncRNA |
| 406978 | MIR199B | 9q34.11 | microRNA 199b | ncRNA |
| 406979 | MIR19A | 13q31.3 | microRNA 19a | ncRNA |
| 406982 | MIR20A | 13q31.3 | microRNA 20a | ncRNA |
| 406991 | MIR21 | 17q23.1 | microRNA 21 | ncRNA |
| 406992 | MIR210 | 11p15.5 | microRNA 210 | ncRNA |
| 406994 | MIR212 | 17p13.3 | microRNA 212 | ncRNA |
| 406995 | MIR181A1 | 1q32.1 | microRNA 181a-1 | ncRNA |
| 406999 | MIR217 | 2p16.1 | microRNA 217 | ncRNA |
| 407003 | MIR219A2 | 9q34.11 | microRNA 219a-2 | ncRNA |
| 407004 | MIR22 | 17p13.3 | microRNA 22 | ncRNA |
| 407006 | MIR221 | Xp11.3 | microRNA 221 | ncRNA |
| 407007 | MIR222 | Xp11.3 | microRNA 222 | ncRNA |
| 407008 | MIR223 | Xq12 | microRNA 223 | ncRNA |
| 407010 | MIR23A | 19p13.12 | microRNA 23a | ncRNA |
| 407012 | MIR24-1 | 9q22.32 | microRNA 24-1 | ncRNA |
| 407013 | MIR24-2 | 19p13.12 | microRNA 24-2 | ncRNA |
| 407015 | MIR26A1 | 3p22.2 | microRNA 26a-1 | ncRNA |
| 407018 | MIR27A | 19p13.12 | microRNA 27a | ncRNA |
| 407019 | MIR27B | 9q22.32 | microRNA 27b | ncRNA |
| 407021 | MIR29A | 7q32.3 | microRNA 29a | ncRNA |
| 407024 | MIR29B1 | 7q32.3 | microRNA 29b-1 | ncRNA |
| 407025 | MIR29B2 | 1q32.2 | microRNA 29b-2 | ncRNA |
| 407026 | MIR29C | 1q32.2 | microRNA 29c | ncRNA |
| 407034 | MIR30E | 1p34.2 | microRNA 30e | ncRNA |
| 407035 | MIR31 | 9p21.3 | microRNA 31 | ncRNA |
| 407036 | MIR32 | 9q31.3 | microRNA 32 | ncRNA |
| 407040 | MIR34A | 1p36.22|1p36.22 | microRNA 34a | ncRNA |
| 407041 | MIR34B | 11q23.1 | microRNA 34b | ncRNA |
| 407042 | MIR34C | 11q23.1 | microRNA 34c | ncRNA |
| 407046 | MIR9-1 | 1q22 | microRNA 9-1 | ncRNA |
| 407047 | MIR9-2 | 5q14.3 | microRNA 9-2 | ncRNA |
| 407051 | MIR9-3 | 15q26.1 | microRNA 9-3 | ncRNA |
| 407053 | MIR96 | 7q32.2 | microRNA 96 | ncRNA |
| 407055 | MIR99A | 21q21.1 | microRNA 99a | ncRNA |
| 407975 | MIR17HG | 13q31.3 | miR-17-92a-1 cluster host gene | ncRNA |
| 408 | ARRB1 | 11q13.4 | arrestin beta 1 | protein-coding |
| 4084 | MXD1 | 2p13.3 | MAX dimerization protein 1 | protein-coding |
| 4086 | SMAD1 | 4q31.21 | SMAD family member 1 | protein-coding |
| 4087 | SMAD2 | 18q21.1 | SMAD family member 2 | protein-coding |
| 4088 | SMAD3 | 15q22.33 | SMAD family member 3 | protein-coding |
| 4089 | SMAD4 | 18q21.2 | SMAD family member 4 | protein-coding |
| 4092 | SMAD7 | 18q21.1 | SMAD family member 7 | protein-coding |
| 4102 | MAGEA3 | Xq28 | MAGE family member A3 | protein-coding |
| 4103 | MAGEA4 | Xq28 | MAGE family member A4 | protein-coding |
| 4137 | MAPT | 17q21.31 | microtubule associated protein tau | protein-coding |
| 414 | ARSD | Xp22.33 | arylsulfatase D | protein-coding |
| 414062 | CCL3L3 | 17q12 | C-C motif chemokine ligand 3 like 3 | protein-coding |
| 414899 | BLID | 11q24.1 | BH3-like motif containing, cell death inducer | protein-coding |
| 415116 | PIM3 | 22q13.33 | Pim-3 proto-oncogene, serine/threonine kinase | protein-coding |
| 4155 | MBP | 18q23 | myelin basic protein | protein-coding |
| 4170 | MCL1 | 1q21.2 | BCL2 family apoptosis regulator | protein-coding |
| 4192 | MDK | 11p11.2 | midkine (neurite growth-promoting factor 2) | protein-coding |
| 4193 | MDM2 | 12q15 | MDM2 proto-oncogene | protein-coding |
| 4194 | MDM4 | 1q32.1 | MDM4, p53 regulator | protein-coding |
| 4200 | ME2 | 18q21.2 | malic enzyme 2 | protein-coding |
| 4208 | MEF2C | 5q14.3 | myocyte enhancer factor 2C | protein-coding |
| 4210 | MEFV | 16p13.3 | MEFV, pyrin innate immunity regulator | protein-coding |
| 4211 | MEIS1 | 2p14 | Meis homeobox 1 | protein-coding |
| 4214 | MAP3K1 | 5q11.2 | mitogen-activated protein kinase kinase kinase 1 | protein-coding |
| 4221 | MEN1 | 11q13.1 | menin 1 | protein-coding |
| 4233 | MET | 7q31.2 | MET proto-oncogene, receptor tyrosine kinase | protein-coding |
| 4240 | MFGE8 | 15q26.1 | milk fat globule-EGF factor 8 protein | protein-coding |
| 4254 | KITLG | 12q21.32 | KIT ligand | protein-coding |
| 4255 | MGMT | 10q26.3 | O-6-methylguanine-DNA methyltransferase | protein-coding |
| 4277 | MICB | 6p21.33 | MHC class I polypeptide-related sequence B | protein-coding |
| 4282 | MIF | 22q11.23 | macrophage migration inhibitory factor (glycosylation-inhibiting factor) | protein-coding |
| 4288 | MKI67 | 10q26.2 | marker of proliferation Ki-67 | protein-coding |
| 4291 | MLF1 | 3q25.32 | myeloid leukemia factor 1 | protein-coding |
| 4292 | MLH1 | 3p22.2 | mutL homolog 1 | protein-coding |
| 4293 | MAP3K9 | 14q24.2 | mitogen-activated protein kinase kinase kinase 9 | protein-coding |
| 4294 | MAP3K10 | 19q13.2 | mitogen-activated protein kinase kinase kinase 10 | protein-coding |
| 4297 | KMT2A | 11q23.3 | lysine methyltransferase 2A | protein-coding |
| 4298 | MLLT1 | 19p13.3 | MLLT1, super elongation complex subunit | protein-coding |
| 4299 | AFF1 | 4q21.3-q22.1 | AF4/FMR2 family member 1 | protein-coding |
| 43 | ACHE | 7q22.1 | acetylcholinesterase (Cartwright blood group) | protein-coding |
| 4300 | MLLT3 | 9p21.3 | MLLT3, super elongation complex subunit | protein-coding |
| 4301 | AFDN | 6q27 | afadin, adherens junction formation factor | protein-coding |
| 4302 | MLLT6 | 17q12 | MLLT6, PHD finger domain containing | protein-coding |
| 4303 | FOXO4 | Xq13.1 | forkhead box O4 | protein-coding |
| 4311 | MME | 3q25.2 | membrane metalloendopeptidase | protein-coding |
| 4313 | MMP2 | 16q12.2 | matrix metallopeptidase 2 | protein-coding |
| 4316 | MMP7 | 11q22.2 | matrix metallopeptidase 7 | protein-coding |
| 4318 | MMP9 | 20q13.12 | matrix metallopeptidase 9 | protein-coding |
| 4330 | MN1 | 22q12.1 | MN1 proto-oncogene, transcriptional regulator | protein-coding |
| 4335 | MNT | 17p13.3 | MAX network transcriptional repressor | protein-coding |
| 4345 | CD200 | 3q13.2 | CD200 molecule | protein-coding |
| 4352 | MPL | 1p34.2 | MPL proto-oncogene, thrombopoietin receptor | protein-coding |
| 4353 | MPO | 17q22 | myeloperoxidase | protein-coding |
| 4363 | ABCC1 | 16p13.11 | ATP binding cassette subfamily C member 1 | protein-coding |
| 440 | ASNS | 7q21.3 | asparagine synthetase (glutamine-hydrolyzing) | protein-coding |
| 442892 | MIR148B | 12q13.13 | microRNA 148b | ncRNA |
| 442900 | MIR326 | 11q13.4 | microRNA 326 | ncRNA |
| 442901 | MIR328 | 16q22.1 | microRNA 328 | ncRNA |
| 442905 | MIR337 | 14q32.2 | microRNA 337 | ncRNA |
| 442906 | MIR338 | 17q25.3 | microRNA 338 | ncRNA |
| 442915 | MIR370 | 14q32.31 | microRNA 370 | ncRNA |
| 442920 | MIR196B | 7p15.2 | microRNA 196b | ncRNA |
| 4436 | MSH2 | 2p21-p16.3 | mutS homolog 2 | protein-coding |
| 4478 | MSN | Xq12 | moesin | protein-coding |
| 4481 | MSR1 | 8p22 | macrophage scavenger receptor 1 | protein-coding |
| 4486 | MST1R | 3p21.31 | macrophage stimulating 1 receptor | protein-coding |
| 4507 | MTAP | 9p21.3 | methylthioadenosine phosphorylase | protein-coding |
| 4508 | ATP6 | - | ATP synthase F0 subunit 6 | protein-coding |
| 4513 | COX2 | - | cytochrome c oxidase subunit II | protein-coding |
| 4515 | MTCP1 | Xq28 | mature T-cell proliferation 1 | protein-coding |
| 4522 | MTHFD1 | 14q23.3 | methylenetetrahydrofolate dehydrogenase, cyclohydrolase and formyltetrahydrofolate synthetase 1 | protein-coding |
| 4524 | MTHFR | 1p36.22 | methylenetetrahydrofolate reductase | protein-coding |
| 4548 | MTR | 1q43 | 5-methyltetrahydrofolate-homocysteine methyltransferase | protein-coding |
| 4552 | MTRR | 5p15.31 | 5-methyltetrahydrofolate-homocysteine methyltransferase reductase | protein-coding |
| 4582 | MUC1 | 1q22 | mucin 1, cell surface associated | protein-coding |
| 4595 | MUTYH | 1p34.1 | mutY DNA glycosylase | protein-coding |
| 4601 | MXI1 | 10q25.2 | MAX interactor 1, dimerization protein | protein-coding |
| 4602 | MYB | 6q23.3 | MYB proto-oncogene, transcription factor | protein-coding |
| 4609 | MYC | 8q24.21 | v-myc avian myelocytomatosis viral oncogene homolog | protein-coding |
| 4613 | MYCN | 2p24.3 | v-myc avian myelocytomatosis viral oncogene neuroblastoma derived homolog | protein-coding |
| 4615 | MYD88 | 3p22.2 | myeloid differentiation primary response 88 | protein-coding |
| 4627 | MYH9 | 22q12.3 | myosin heavy chain 9 | protein-coding |
| 4629 | MYH11 | 16p13.11 | myosin heavy chain 11 | protein-coding |
| 4666 | NACA | 12q13.3 | nascent polypeptide-associated complex alpha subunit | protein-coding |
| 4671 | NAIP | 5q13.2 | NLR family apoptosis inhibitory protein | protein-coding |
| 468 | ATF4 | 22q13.1 | activating transcription factor 4 | protein-coding |
| 4680 | CEACAM6 | 19q13.2 | carcinoembryonic antigen related cell adhesion molecule 6 | protein-coding |
| 4683 | NBN | 8q21.3 | nibrin | protein-coding |
| 4684 | NCAM1 | 11q23.2 | neural cell adhesion molecule 1 | protein-coding |
| 4691 | NCL | 2q37.1 | nucleolin | protein-coding |
| 472 | ATM | 11q22.3 | ATM serine/threonine kinase | protein-coding |
| 4739 | NEDD9 | 6p24.2 | neural precursor cell expressed, developmentally down-regulated 9 | protein-coding |
| 4763 | NF1 | 17q11.2 | neurofibromin 1 | protein-coding |
| 4772 | NFATC1 | 18q23 | nuclear factor of activated T-cells 1 | protein-coding |
| 4773 | NFATC2 | 20q13.2 | nuclear factor of activated T-cells 2 | protein-coding |
| 4778 | NFE2 | 12q13.13 | nuclear factor, erythroid 2 | protein-coding |
| 4780 | NFE2L2 | 2q31.2 | nuclear factor, erythroid 2 like 2 | protein-coding |
| 4784 | NFIX | 19p13.13 | nuclear factor I X | protein-coding |
| 4790 | NFKB1 | 4q24 | nuclear factor kappa B subunit 1 | protein-coding |
| 4791 | NFKB2 | 10q24.32 | nuclear factor kappa B subunit 2 | protein-coding |
| 4794 | NFKBIE | 6p21.1 | NFKB inhibitor epsilon | protein-coding |
| 4801 | NFYB | 12q23.3 | nuclear transcription factor Y subunit beta | protein-coding |
| 4803 | NGF | 1p13.2 | nerve growth factor | protein-coding |
| 4804 | NGFR | 17q21.33 | nerve growth factor receptor | protein-coding |
| 4821 | NKX2-2 | 20p11.22 | NK2 homeobox 2 | protein-coding |
| 4824 | NKX3-1 | 8p21.2 | NK3 homeobox 1 | protein-coding |
| 4826 | NNAT | 20q11.23 | neuronatin | protein-coding |
| 4830 | NME1 | 17q21.33 | NME/NM23 nucleoside diphosphate kinase 1 | protein-coding |
| 4831 | NME2 | 17q21.33 | NME/NM23 nucleoside diphosphate kinase 2 | protein-coding |
| 4837 | NNMT | 11q23.2 | nicotinamide N-methyltransferase | protein-coding |
| 4843 | NOS2 | 17q11.2 | nitric oxide synthase 2 | protein-coding |
| 4846 | NOS3 | 7q36.1 | nitric oxide synthase 3 | protein-coding |
| 4849 | CNOT3 | 19q13.42 | CCR4-NOT transcription complex subunit 3 | protein-coding |
| 4851 | NOTCH1 | 9q34.3 | notch 1 | protein-coding |
| 4853 | NOTCH2 | 1p12 | notch 2 | protein-coding |
| 4854 | NOTCH3 | 19p13.12 | notch 3 | protein-coding |
| 4855 | NOTCH4 | 6p21.32 | notch 4 | protein-coding |
| 4856 | NOV | 8q24.12 | nephroblastoma overexpressed | protein-coding |
| 4869 | NPM1 | 5q35.1 | nucleophosmin | protein-coding |
| 4893 | NRAS | 1p13.2 | neuroblastoma RAS viral oncogene homolog | protein-coding |
| 4907 | NT5E | 6q14.3 | 5'-nucleotidase ecto | protein-coding |
| 4914 | NTRK1 | 1q23.1 | neurotrophic receptor tyrosine kinase 1 | protein-coding |
| 4916 | NTRK3 | 15q25.3 | neurotrophic receptor tyrosine kinase 3 | protein-coding |
| 4919 | ROR1 | 1p31.3 | receptor tyrosine kinase like orphan receptor 1 | protein-coding |
| 4922 | NTS | 12q21.31 | neurotensin | protein-coding |
| 4923 | NTSR1 | 20q13.33 | neurotensin receptor 1 | protein-coding |
| 4926 | NUMA1 | 11q13.4 | nuclear mitotic apparatus protein 1 | protein-coding |
| 4927 | NUP88 | 17p13.2 | nucleoporin 88 | protein-coding |
| 4928 | NUP98 | 11p15.4 | nucleoporin 98 | protein-coding |
| 4940 | OAS3 | 12q24.13 | 2'-5'-oligoadenylate synthetase 3 | protein-coding |
| 494324 | MIR375 | 2q35 | microRNA 375 | ncRNA |
| 494327 | MIR378A | 5q32 | microRNA 378a | ncRNA |
| 494336 | MIR424 | Xq26.3 | microRNA 424 | ncRNA |
| 4946 | OAZ1 | 19p13.3 | ornithine decarboxylase antizyme 1 | protein-coding |
| 4968 | OGG1 | 3p25.3 | 8-oxoguanine DNA glycosylase | protein-coding |
| 4982 | TNFRSF11B | 8q24.12 | TNF receptor superfamily member 11b | protein-coding |
| 4986 | OPRK1 | 8q11.23 | opioid receptor kappa 1 | protein-coding |
| 5004 | ORM1 | 9q32 | orosomucoid 1 | protein-coding |
| 5023 | P2RX1 | 17p13.2 | purinergic receptor P2X 1 | protein-coding |
| 5026 | P2RX5 | 17p13.2 | purinergic receptor P2X 5 | protein-coding |
| 5027 | P2RX7 | 12q24.31 | purinergic receptor P2X 7 | protein-coding |
| 5037 | PEBP1 | 12q24.23 | phosphatidylethanolamine binding protein 1 | protein-coding |
| 50486 | G0S2 | 1q32.2 | G0/G1 switch 2 | protein-coding |
| 50507 | NOX4 | 11q14.3 | NADPH oxidase 4 | protein-coding |
| 5054 | SERPINE1 | 7q22.1 | serpin family E member 1 | protein-coding |
| 506 | ATP5B | 12q13.3 | ATP synthase, H+ transporting, mitochondrial F1 complex, beta polypeptide | protein-coding |
| 50615 | IL21R | 16p12.1 | interleukin 21 receptor | protein-coding |
| 50616 | IL22 | 12q15 | interleukin 22 | protein-coding |
| 5071 | PARK2 | 6q26 | parkin RBR E3 ubiquitin protein ligase | protein-coding |
| 5074 | PAWR | 12q21.2 | pro-apoptotic WT1 regulator | protein-coding |
| 5078 | PAX4 | 7q32.1 | paired box 4 | protein-coding |
| 5079 | PAX5 | 9p13.2 | paired box 5 | protein-coding |
| 50802 | IGK | 2p11.2 | immunoglobulin kappa locus | protein-coding |
| 50859 | SPOCK3 | 4q32.3 | SPARC/osteonectin, cwcv and kazal like domains proteoglycan 3 | protein-coding |
| 5087 | PBX1 | 1q23.3 | PBX homeobox 1 | protein-coding |
| 5090 | PBX3 | 9q33.3 | PBX homeobox 3 | protein-coding |
| 5094 | PCBP2 | 12q13.13 | poly(rC) binding protein 2 | protein-coding |
| 50943 | FOXP3 | Xp11.23 | forkhead box P3 | protein-coding |
| 51024 | FIS1 | 7q22.1 | fission, mitochondrial 1 | protein-coding |
| 51053 | GMNN | 6p22.3 | geminin, DNA replication inhibitor | protein-coding |
| 5108 | PCM1 | 8p22 | pericentriolar material 1 | protein-coding |
| 51094 | ADIPOR1 | 1q32.1 | adiponectin receptor 1 | protein-coding |
| 51119 | SBDS | 7q11.21 | SBDS ribosome assembly guanine nucleotide exchange factor | protein-coding |
| 51176 | LEF1 | 4q25 | lymphoid enhancer binding factor 1 | protein-coding |
| 51193 | ZNF639 | 3q26.33 | zinc finger protein 639 | protein-coding |
| 51203 | NUSAP1 | 15q15.1 | nucleolar and spindle associated protein 1 | protein-coding |
| 51232 | CRIM1 | 2p22.2 | cysteine rich transmembrane BMP regulator 1 | protein-coding |
| 51237 | MZB1 | 5q31.2 | marginal zone B and B1 cell specific protein | protein-coding |
| 51251 | NT5C3A | 7p14.3 | 5'-nucleotidase, cytosolic IIIA | protein-coding |
| 51271 | UBAP1 | 9p13.3 | ubiquitin associated protein 1 | protein-coding |
| 51274 | KLF3 | 4p14 | Kruppel like factor 3 | protein-coding |
| 51278 | IER5 | 1q25.3 | immediate early response 5 | protein-coding |
| 51284 | TLR7 | Xp22.2 | toll like receptor 7 | protein-coding |
| 51292 | GMPR2 | 14q12 | guanosine monophosphate reductase 2 | protein-coding |
| 5133 | PDCD1 | 2q37.3 | programmed cell death 1 | protein-coding |
| 5134 | PDCD2 | 6q27 | programmed cell death 2 | protein-coding |
| 51341 | ZBTB7A | 19p13.3 | zinc finger and BTB domain containing 7A | protein-coding |
| 51384 | WNT16 | 7q31.31 | Wnt family member 16 | protein-coding |
| 5140 | PDE3B | 11p15.2 | phosphodiesterase 3B | protein-coding |
| 5141 | PDE4A | 19p13.2 | phosphodiesterase 4A | protein-coding |
| 5142 | PDE4B | 1p31.3 | phosphodiesterase 4B | protein-coding |
| 5144 | PDE4D | 5q11.2-q12.1 | phosphodiesterase 4D | protein-coding |
| 515 | ATP5F1 | 1p13.2 | ATP synthase, H+ transporting, mitochondrial Fo complex subunit B1 | protein-coding |
| 51510 | CHMP5 | 9p13.3 | charged multivesicular body protein 5 | protein-coding |
| 51523 | CXXC5 | 5q31.2 | CXXC finger protein 5 | protein-coding |
| 51547 | SIRT7 | 17q25.3 | sirtuin 7 | protein-coding |
| 5156 | PDGFRA | 4q12 | platelet derived growth factor receptor alpha | protein-coding |
| 51561 | IL23A | 12q13.3 | interleukin 23 subunit alpha | protein-coding |
| 5159 | PDGFRB | 5q32 | platelet derived growth factor receptor beta | protein-coding |
| 51593 | SRRT | 7q22.1 | serrate, RNA effector molecule | protein-coding |
| 51617 | HMP19 | 5q35.2 | HMP19 protein | protein-coding |
| 5163 | PDK1 | 2q31.1 | pyruvate dehydrogenase kinase 1 | protein-coding |
| 5168 | ENPP2 | 8q24.12 | ectonucleotide pyrophosphatase/phosphodiesterase 2 | protein-coding |
| 51684 | SUFU | 10q24.32 | SUFU negative regulator of hedgehog signaling | protein-coding |
| 5170 | PDPK1 | 16p13.3 | 3-phosphoinositide dependent protein kinase 1 | protein-coding |
| 51738 | GHRL | 3p25.3 | ghrelin and obestatin prepropeptide | protein-coding |
| 51741 | WWOX | 16q23.1-q23.2 | WW domain containing oxidoreductase | protein-coding |
| 51742 | ARID4B | 1q42.3 | AT-rich interaction domain 4B | protein-coding |
| 51744 | CD244 | 1q23.3 | CD244 molecule | protein-coding |
| 5175 | PECAM1 | 17q23.3 | platelet and endothelial cell adhesion molecule 1 | protein-coding |
| 51780 | KDM3B | 5q31.2 | lysine demethylase 3B | protein-coding |
| 5196 | PF4 | 4q13.3 | platelet factor 4 | protein-coding |
| 52 | ACP1 | 2p25.3 | acid phosphatase 1, soluble | protein-coding |
| 5226 | PGD | 1p36.22 | phosphogluconate dehydrogenase | protein-coding |
| 5228 | PGF | 14q24.3 | placental growth factor | protein-coding |
| 5241 | PGR | 11q22.1 | progesterone receptor | protein-coding |
| 5243 | ABCB1 | 7q21.12 | ATP binding cassette subfamily B member 1 | protein-coding |
| 5245 | PHB | 17q21.33 | prohibitin | protein-coding |
| 5265 | SERPINA1 | 14q32.13 | serpin family A member 1 | protein-coding |
| 5272 | SERPINB9 | 6p25.2 | serpin family B member 9 | protein-coding |
| 5286 | PIK3C2A | 11p15.1 | phosphatidylinositol-4-phosphate 3-kinase catalytic subunit type 2 alpha | protein-coding |
| 5289 | PIK3C3 | 18q12.3 | phosphatidylinositol 3-kinase catalytic subunit type 3 | protein-coding |
| 5290 | PIK3CA | 3q26.32 | phosphatidylinositol-4,5-bisphosphate 3-kinase catalytic subunit alpha | protein-coding |
| 5292 | PIM1 | 6p21.2 | Pim-1 proto-oncogene, serine/threonine kinase | protein-coding |
| 5293 | PIK3CD | 1p36.22 | phosphatidylinositol-4,5-bisphosphate 3-kinase catalytic subunit delta | protein-coding |
| 5294 | PIK3CG | 7q22.3 | phosphatidylinositol-4,5-bisphosphate 3-kinase catalytic subunit gamma | protein-coding |
| 5295 | PIK3R1 | 5q13.1 | phosphoinositide-3-kinase regulatory subunit 1 | protein-coding |
| 5296 | PIK3R2 | 19p13.11 | phosphoinositide-3-kinase regulatory subunit 2 | protein-coding |
| 5300 | PIN1 | 19p13.2 | peptidylprolyl cis/trans isomerase, NIMA-interacting 1 | protein-coding |
| 5305 | PIP4K2A | 10p12.2 | phosphatidylinositol-5-phosphate 4-kinase type 2 alpha | protein-coding |
| 5315 | PKM | 15q23 | pyruvate kinase, muscle | protein-coding |
| 5321 | PLA2G4A | 1q31.1 | phospholipase A2 group IVA | protein-coding |
| 5324 | PLAG1 | 8q12.1 | PLAG1 zinc finger | protein-coding |
| 5326 | PLAGL2 | 20q11.21 | PLAG1 like zinc finger 2 | protein-coding |
| 5327 | PLAT | 8p11.21 | plasminogen activator, tissue type | protein-coding |
| 5329 | PLAUR | 19q13.31 | plasminogen activator, urokinase receptor | protein-coding |
| 5333 | PLCD1 | 3p22.2 | phospholipase C delta 1 | protein-coding |
| 53335 | BCL11A | 2p16.1 | B-cell CLL/lymphoma 11A | protein-coding |
| 5335 | PLCG1 | 20q12 | phospholipase C gamma 1 | protein-coding |
| 5336 | PLCG2 | 16q23.3 | phospholipase C gamma 2 | protein-coding |
| 5337 | PLD1 | 3q26.31 | phospholipase D1 | protein-coding |
| 5347 | PLK1 | 16p12.2 | polo like kinase 1 | protein-coding |
| 5359 | PLSCR1 | 3q24 | phospholipid scramblase 1 | protein-coding |
| 53615 | MBD3 | 19p13.3 | methyl-CpG binding domain protein 3 | protein-coding |
| 53637 | S1PR5 | 19p13.2 | sphingosine-1-phosphate receptor 5 | protein-coding |
| 5366 | PMAIP1 | 18q21.32 | phorbol-12-myristate-13-acetate-induced protein 1 | protein-coding |
| 5371 | PML | 15q24.1 | promyelocytic leukemia | protein-coding |
| 53831 | GPR84 | 12q13.13 | G protein-coupled receptor 84 | protein-coding |
| 53947 | A4GALT | 22q13.2 | alpha 1,4-galactosyltransferase | protein-coding |
| 54106 | TLR9 | 3p21.2 | toll like receptor 9 | protein-coding |
| 5414 | Sep-04 | 17q22 | septin 4 | protein-coding |
| 5420 | PODXL | 7q32.3 | podocalyxin like | protein-coding |
| 54361 | WNT4 | 1p36.12 | Wnt family member 4 | protein-coding |
| 54386 | TERF2IP | 16q23.1 | TERF2 interacting protein | protein-coding |
| 54414 | SIAE | 11q24.2 | sialic acid acetylesterase | protein-coding |
| 5443 | POMC | 2p23.3 | proopiomelanocortin | protein-coding |
| 5444 | PON1 | 7q21.3 | paraoxonase 1 | protein-coding |
| 54457 | TAF7L | Xq22.1 | TATA-box binding protein associated factor 7 like | protein-coding |
| 5447 | POR | 7q11.23 | cytochrome p450 oxidoreductase | protein-coding |
| 545 | ATR | 3q23 | ATR serine/threonine kinase | protein-coding |
| 5450 | POU2AF1 | 11q23.1 | POU class 2 associating factor 1 | protein-coding |
| 5451 | POU2F1 | 1q24.2 | POU class 2 homeobox 1 | protein-coding |
| 5452 | POU2F2 | 19q13.2 | POU class 2 homeobox 2 | protein-coding |
| 54567 | DLL4 | 15q15.1 | delta like canonical Notch ligand 4 | protein-coding |
| 5457 | POU4F1 | 13q31.1 | POU class 4 homeobox 1 | protein-coding |
| 546 | ATRX | Xq21.1 | ATRX, chromatin remodeler | protein-coding |
| 5460 | POU5F1 | 6p21.33 | POU class 5 homeobox 1 | protein-coding |
| 5465 | PPARA | 22q13.31 | peroxisome proliferator activated receptor alpha | protein-coding |
| 54658 | UGT1A1 | 2q37.1 | UDP glucuronosyltransferase family 1 member A1 | protein-coding |
| 5468 | PPARG | 3p25.2 | peroxisome proliferator activated receptor gamma | protein-coding |
| 54739 | XAF1 | 17p13.1 | XIAP associated factor 1 | protein-coding |
| 54790 | TET2 | 4q24 | tet methylcytosine dioxygenase 2 | protein-coding |
| 54806 | AHI1 | 6q23.3 | Abelson helper integration site 1 | protein-coding |
| 54820 | NDE1 | 16p13.11 | nudE neurodevelopment protein 1 | protein-coding |
| 54878 | DPP8 | 15q22.31 | dipeptidyl peptidase 8 | protein-coding |
| 54880 | BCOR | Xp11.4 | BCL6 corepressor | protein-coding |
| 54882 | ANKHD1 | 5q31.3 | ankyrin repeat and KH domain containing 1 | protein-coding |
| 54970 | TTC12 | 11q23.2 | tetratricopeptide repeat domain 12 | protein-coding |
| 55120 | FANCL | 2p16.1 | Fanconi anemia complementation group L | protein-coding |
| 55124 | PIWIL2 | 8p21.3 | piwi like RNA-mediated gene silencing 2 | protein-coding |
| 55145 | THAP1 | 8p11.21 | THAP domain containing 1 | protein-coding |
| 55177 | RMDN3 | 15q15.1 | regulator of microtubule dynamics 3 | protein-coding |
| 5520 | PPP2R2A | 8p21.2 | protein phosphatase 2 regulatory subunit Balpha | protein-coding |
| 5521 | PPP2R2B | 5q32 | protein phosphatase 2 regulatory subunit Bbeta | protein-coding |
| 55213 | RCBTB1 | 13q14.2 | RCC1 and BTB domain containing protein 1 | protein-coding |
| 5524 | PTPA | 9q34.11 | protein phosphatase 2 phosphatase activator | protein-coding |
| 5525 | PPP2R5A | 1q32.3 | protein phosphatase 2 regulatory subunit B'alpha | protein-coding |
| 55250 | ELP2 | 18q12.2 | elongator acetyltransferase complex subunit 2 | protein-coding |
| 55252 | ASXL2 | 2p23.3 | additional sex combs like 2, transcriptional regulator | protein-coding |
| 5527 | PPP2R5C | 14q32.31 | protein phosphatase 2 regulatory subunit B'gamma | protein-coding |
| 55270 | NUDT15 | 13q14.2 | nudix hydrolase 15 | protein-coding |
| 55294 | FBXW7 | 4q31.3 | F-box and WD repeat domain containing 7 | protein-coding |
| 5531 | PPP4C | 16p11.2 | protein phosphatase 4 catalytic subunit | protein-coding |
| 55332 | DRAM1 | 12q23.2 | DNA damage regulated autophagy modulator 1 | protein-coding |
| 55359 | STYK1 | 12p13.2 | serine/threonine/tyrosine kinase 1 | protein-coding |
| 55363 | HEMGN | 9q22.33 | hemogen | protein-coding |
| 55384 | MEG3 | 14q32.2 | maternally expressed 3 (non-protein coding) | ncRNA |
| 5549 | PRELP | 1q32.1 | proline and arginine rich end leucine rich repeat protein | protein-coding |
| 5550 | PREP | 6q21 | prolyl endopeptidase | protein-coding |
| 55500 | ETNK1 | 12p12.1 | ethanolamine kinase 1 | protein-coding |
| 55503 | TRPV6 | 7q34 | transient receptor potential cation channel subfamily V member 6 | protein-coding |
| 5551 | PRF1 | 10q22.1 | perforin 1 | protein-coding |
| 55510 | DDX43 | 6q13 | DEAD-box helicase 43 | protein-coding |
| 55512 | SMPD3 | 16q22.1 | sphingomyelin phosphodiesterase 3 | protein-coding |
| 55553 | SOX6 | 11p15.2 | SRY-box 6 | protein-coding |
| 5562 | PRKAA1 | 5p13.1 | protein kinase AMP-activated catalytic subunit alpha 1 | protein-coding |
| 5564 | PRKAB1 | 12q24.23 | protein kinase AMP-activated non-catalytic subunit beta 1 | protein-coding |
| 5566 | PRKACA | 19p13.12 | protein kinase cAMP-activated catalytic subunit alpha | protein-coding |
| 55743 | CHFR | 12q24.33 | checkpoint with forkhead and ring finger domains | protein-coding |
| 55749 | CCAR1 | 10q21.3 | cell division cycle and apoptosis regulator 1 | protein-coding |
| 55760 | DHX32 | 10q26.2 | DEAH-box helicase 32 (putative) | protein-coding |
| 5578 | PRKCA | 17q24.2 | protein kinase C alpha | protein-coding |
| 5579 | PRKCB | 16p12.2-p12.1 | protein kinase C beta | protein-coding |
| 558 | AXL | 19q13.2 | AXL receptor tyrosine kinase | protein-coding |
| 5580 | PRKCD | 3p21.1 | protein kinase C delta | protein-coding |
| 5581 | PRKCE | 2p21 | protein kinase C epsilon | protein-coding |
| 5583 | PRKCH | 14q23.1 | protein kinase C eta | protein-coding |
| 55854 | ZC3H15 | 2q32.1 | zinc finger CCCH-type containing 15 | protein-coding |
| 55859 | BEX1 | Xq22.1|Xq22 | brain expressed X-linked 1 | protein-coding |
| 55869 | HDAC8 | Xq13.1 | histone deacetylase 8 | protein-coding |
| 55872 | PBK | 8p21.1 | PDZ binding kinase | protein-coding |
| 5588 | PRKCQ | 10p15.1 | protein kinase C theta | protein-coding |
| 55904 | KMT2E | 7q22.3 | lysine methyltransferase 2E | protein-coding |
| 5591 | PRKDC | 8q11.21 | protein kinase, DNA-activated, catalytic polypeptide | protein-coding |
| 5594 | MAPK1 | 22q11.22 | mitogen-activated protein kinase 1 | protein-coding |
| 5595 | MAPK3 | 16p11.2 | mitogen-activated protein kinase 3 | protein-coding |
| 5598 | MAPK7 | 17p11.2 | mitogen-activated protein kinase 7 | protein-coding |
| 5599 | MAPK8 | 10q11.22 | mitogen-activated protein kinase 8 | protein-coding |
| 56001 | NXF2 | Xq22.1 | nuclear RNA export factor 2 | protein-coding |
| 5601 | MAPK9 | 5q35.3 | mitogen-activated protein kinase 9 | protein-coding |
| 5603 | MAPK13 | 6p21.31 | mitogen-activated protein kinase 13 | protein-coding |
| 5604 | MAP2K1 | 15q22.31 | mitogen-activated protein kinase kinase 1 | protein-coding |
| 5609 | MAP2K7 | 19p13.2 | mitogen-activated protein kinase kinase 7 | protein-coding |
| 5610 | EIF2AK2 | 2p22.2 | eukaryotic translation initiation factor 2 alpha kinase 2 | protein-coding |
| 5621 | PRNP | 20p13 | prion protein | protein-coding |
| 56254 | RNF20 | 9q31.1 | ring finger protein 20 | protein-coding |
| 56302 | TRPV5 | 7q34 | transient receptor potential cation channel subfamily V member 5 | protein-coding |
| 5641 | LGMN | 14q32.12 | legumain | protein-coding |
| 5657 | PRTN3 | 19p13.3 | proteinase 3 | protein-coding |
| 56616 | DIABLO | 12q24.31 | diablo IAP-binding mitochondrial protein | protein-coding |
| 56675 | NRIP3 | 11p15.4 | nuclear receptor interacting protein 3 | protein-coding |
| 567 | B2M | 15q21.1 | beta-2-microglobulin | protein-coding |
| 56899 | ANKS1B | 12q23.1 | ankyrin repeat and sterile alpha motif domain containing 1B | protein-coding |
| 56917 | MEIS3 | 19q13.32 | Meis homeobox 3 | protein-coding |
| 56925 | LXN | 3q25.32 | latexin | protein-coding |
| 56950 | SMYD2 | 1q32.3 | SET and MYND domain containing 2 | protein-coding |
| 5697 | PYY | 17q21.31 | peptide YY | protein-coding |
| 56979 | PRDM9 | 5p14.2 | PR/SET domain 9 | protein-coding |
| 5698 | PSMB9 | 6p21.32 | proteasome subunit beta 9 | protein-coding |
| 56983 | POGLUT1 | 3q13.33 | protein O-glucosyltransferase 1 | protein-coding |
| 57007 | ACKR3 | 2q37.3 | atypical chemokine receptor 3 | protein-coding |
| 57019 | CIAPIN1 | 16q21 | cytokine induced apoptosis inhibitor 1 | protein-coding |
| 57099 | AVEN | 15q14 | apoptosis and caspase activation inhibitor | protein-coding |
| 571 | BACH1 | 21q21.3 | BTB domain and CNC homolog 1 | protein-coding |
| 57103 | TIGAR | 12p13.32 | TP53 induced glycolysis regulatory phosphatase | protein-coding |
| 57118 | CAMK1D | 10p13 | calcium/calmodulin dependent protein kinase ID | protein-coding |
| 57121 | LPAR5 | 12p13.31 | lysophosphatidic acid receptor 5 | protein-coding |
| 57142 | RTN4 | 2p16.1 | reticulon 4 | protein-coding |
| 57167 | SALL4 | 20q13.2 | spalt like transcription factor 4 | protein-coding |
| 57178 | ZMIZ1 | 10q22.3 | zinc finger MIZ-type containing 1 | protein-coding |
| 57215 | THAP11 | 16q22.1 | THAP domain containing 11 | protein-coding |
| 5724 | PTAFR | 1p35.3 | platelet activating factor receptor | protein-coding |
| 5728 | PTEN | 10q23.31 | phosphatase and tensin homolog | protein-coding |
| 573 | BAG1 | 9p13.3 | BCL2 associated athanogene 1 | protein-coding |
| 57379 | AICDA | 12p13.31 | activation induced cytidine deaminase | protein-coding |
| 5739 | PTGIR | 19q13.32 | prostaglandin I2 (prostacyclin) receptor (IP) | protein-coding |
| 574028 | CLLU1 | 12q22 | chronic lymphocytic leukemia up-regulated 1 | protein-coding |
| 574029 | DUSP5P1 | 1q42.13 | dual specificity phosphatase 5 pseudogene 1 | pseudo |
| 5742 | PTGS1 | 9q33.2 | prostaglandin-endoperoxide synthase 1 | protein-coding |
| 5743 | PTGS2 | 1q31.1 | prostaglandin-endoperoxide synthase 2 | protein-coding |
| 5744 | PTHLH | 12p11.22 | parathyroid hormone like hormone | protein-coding |
| 574411 | MIR451A | 17q11.2 | microRNA 451a | ncRNA |
| 574453 | MIR495 | 14q32.31 | microRNA 495 | ncRNA |
| 574455 | MIR193B | 16p13.12 | microRNA 193b | ncRNA |
| 57447 | NDRG2 | 14q11.2 | NDRG family member 2 | protein-coding |
| 57448 | BIRC6 | 2p22.3 | baculoviral IAP repeat containing 6 | protein-coding |
| 574501 | MIR499A | 20q11.22 | microRNA 499a | ncRNA |
| 574504 | MIR502 | Xp11.23 | microRNA 502 | ncRNA |
| 5747 | PTK2 | 8q24.3 | protein tyrosine kinase 2 | protein-coding |
| 57521 | RPTOR | 17q25.3 | regulatory associated protein of MTOR complex 1 | protein-coding |
| 5754 | PTK7 | 6p21.1 | protein tyrosine kinase 7 (inactive) | protein-coding |
| 57569 | ARHGAP20 | 11q22.3-q23.1 | Rho GTPase activating protein 20 | protein-coding |
| 57575 | PCDH10 | 4q28.3 | protocadherin 10 | protein-coding |
| 57591 | MKL1 | 22q13.1-q13.2 | megakaryoblastic leukemia (translocation) 1 | protein-coding |
| 57650 | KIAA1524 | 3q13.13 | KIAA1524 | protein-coding |
| 5770 | PTPN1 | 20q13.13 | protein tyrosine phosphatase, non-receptor type 1 | protein-coding |
| 5771 | PTPN2 | 18p11.21 | protein tyrosine phosphatase, non-receptor type 2 | protein-coding |
| 57761 | TRIB3 | 20p13 | tribbles pseudokinase 3 | protein-coding |
| 5777 | PTPN6 | 12p13.31 | protein tyrosine phosphatase, non-receptor type 6 | protein-coding |
| 578 | BAK1 | 6p21.31 | BCL2 antagonist/killer 1 | protein-coding |
| 5781 | PTPN11 | 12q24.13 | protein tyrosine phosphatase, non-receptor type 11 | protein-coding |
| 57817 | HAMP | 19q13.12 | hepcidin antimicrobial peptide | protein-coding |
| 5788 | PTPRC | 1q31.3-q32.1 | protein tyrosine phosphatase, receptor type C | protein-coding |
| 5793 | PTPRG | 3p14.2 | protein tyrosine phosphatase, receptor type G | protein-coding |
| 5800 | PTPRO | 12p12.3|12p13-p12 | protein tyrosine phosphatase, receptor type O | protein-coding |
| 581 | BAX | 19q13.33 | BCL2 associated X, apoptosis regulator | protein-coding |
| 58155 | PTBP2 | 1p21.3 | polypyrimidine tract binding protein 2 | protein-coding |
| 5817 | PVR | 19q13.31 | poliovirus receptor | protein-coding |
| 5819 | NECTIN2 | 19q13.32 | nectin cell adhesion molecule 2 | protein-coding |
| 5820 | PVT1 | 8q24.21 | Pvt1 oncogene (non-protein coding) | ncRNA |
| 58508 | KMT2C | 7q36.1 | lysine methyltransferase 2C | protein-coding |
| 586 | BCAT1 | 12p12.1 | branched chain amino acid transaminase 1 | protein-coding |
| 5879 | RAC1 | 7p22.1 | ras-related C3 botulinum toxin substrate 1 (rho family, small GTP binding protein Rac1) | protein-coding |
| 5880 | RAC2 | 22q13.1 | ras-related C3 botulinum toxin substrate 2 (rho family, small GTP binding protein Rac2) | protein-coding |
| 5881 | RAC3 | 17q25.3 | ras-related C3 botulinum toxin substrate 3 (rho family, small GTP binding protein Rac3) | protein-coding |
| 5888 | RAD51 | 15q15.1 | RAD51 recombinase | protein-coding |
| 5893 | RAD52 | 12p13.33 | RAD52 homolog, DNA repair protein | protein-coding |
| 5894 | RAF1 | 3p25.2 | Raf-1 proto-oncogene, serine/threonine kinase | protein-coding |
| 5896 | RAG1 | 11p12 | recombination activating 1 | protein-coding |
| 5897 | RAG2 | 11p12 | recombination activating 2 | protein-coding |
| 5898 | RALA | 7p14.1 | RAS like proto-oncogene A | protein-coding |
| 5903 | RANBP2 | 2q13 | RAN binding protein 2 | protein-coding |
| 5906 | RAP1A | 1p13.2 | RAP1A, member of RAS oncogene family | protein-coding |
| 59067 | IL21 | 4q27 | interleukin 21 | protein-coding |
| 5909 | RAP1GAP | 1p36.12 | RAP1 GTPase activating protein | protein-coding |
| 5914 | RARA | 17q21.2 | retinoic acid receptor alpha | protein-coding |
| 5915 | RARB | 3p24.2 | retinoic acid receptor beta | protein-coding |
| 5916 | RARG | 12q13.13 | retinoic acid receptor gamma | protein-coding |
| 5921 | RASA1 | 5q14.3 | RAS p21 protein activator 1 | protein-coding |
| 5923 | RASGRF1 | 15q25.1 | Ras protein specific guanine nucleotide releasing factor 1 | protein-coding |
| 5925 | RB1 | 13q14.2 | RB transcriptional corepressor 1 | protein-coding |
| 5926 | ARID4A | 14q23.1 | AT-rich interaction domain 4A | protein-coding |
| 59307 | SIGIRR | 11p15.5 | single Ig and TIR domain containing | protein-coding |
| 5932 | RBBP8 | 18q11.2 | RB binding protein 8, endonuclease | protein-coding |
| 59339 | PLEKHA2 | 8p11.22 | pleckstrin homology domain containing A2 | protein-coding |
| 595 | CCND1 | 11q13.3 | cyclin D1 | protein-coding |
| 596 | BCL2 | 18q21.33 | BCL2, apoptosis regulator | protein-coding |
| 5962 | RDX | 11q22.3 | radixin | protein-coding |
| 5966 | REL | 2p16.1 | REL proto-oncogene, NF-kB subunit | protein-coding |
| 597 | BCL2A1 | 15q25.1 | BCL2 related protein A1 | protein-coding |
| 5970 | RELA | 11q13.1 | RELA proto-oncogene, NF-kB subunit | protein-coding |
| 5971 | RELB | 19q13.32 | RELB proto-oncogene, NF-kB subunit | protein-coding |
| 5972 | REN | 1q32.1 | renin | protein-coding |
| 598 | BCL2L1 | 20q11.21 | BCL2 like 1 | protein-coding |
| 5981 | RFC1 | 4p14 | replication factor C subunit 1 | protein-coding |
| 599 | BCL2L2 | 14q11.2 | BCL2 like 2 | protein-coding |
| 60 | ACTB | 7p22.1 | actin beta | protein-coding |
| 6007 | RHD | 1p36.11 | Rh blood group D antigen | protein-coding |
| 6016 | RIT1 | 1q22 | Ras like without CAAX 1 | protein-coding |
| 602 | BCL3 | 19q13.32 | B-cell CLL/lymphoma 3 | protein-coding |
| 604 | BCL6 | 3q27.3 | B-cell CLL/lymphoma 6 | protein-coding |
| 6046 | BRD2 | 6p21.32 | bromodomain containing 2 | protein-coding |
| 60468 | BACH2 | 6q15 | BTB domain and CNC homolog 2 | protein-coding |
| 60681 | FKBP10 | 17q21.2 | FK506 binding protein 10 | protein-coding |
| 60682 | SMAP1 | 6q13 | small ArfGAP 1 | protein-coding |
| 608 | TNFRSF17 | 16p13.13 | TNF receptor superfamily member 17 | protein-coding |
| 6093 | ROCK1 | 18q11.1 | Rho associated coiled-coil containing protein kinase 1 | protein-coding |
| 6098 | ROS1 | 6q22.1 | ROS proto-oncogene 1, receptor tyrosine kinase | protein-coding |
| 6125 | RPL5 | 1p22.1 | ribosomal protein L5 | protein-coding |
| 613 | BCR | 22q11.23 | BCR, RhoGEF and GTPase activating protein | protein-coding |
| 6134 | RPL10 | Xq28 | ribosomal protein L10 | protein-coding |
| 6194 | RPS6 | 9p22.1 | ribosomal protein S6 | protein-coding |
| 619554 | MIR486-1 | 8p11.21 | microRNA 486-1 | ncRNA |
| 6196 | RPS6KA2 | 6q27 | ribosomal protein S6 kinase A2 | protein-coding |
| 6198 | RPS6KB1 | 17q23.1 | ribosomal protein S6 kinase B1 | protein-coding |
| 6208 | RPS14 | 5q33.1 | ribosomal protein S14 | protein-coding |
| 6236 | RRAD | 16q22.1 | RRAD, Ras related glycolysis inhibitor and calcium channel regulator | protein-coding |
| 6256 | RXRA | 9q34.2 | retinoid X receptor alpha | protein-coding |
| 627 | BDNF | 11p14.1 | brain derived neurotrophic factor | protein-coding |
| 6279 | S100A8 | 1q21.3 | S100 calcium binding protein A8 | protein-coding |
| 6280 | S100A9 | 1q21.3 | S100 calcium binding protein A9 | protein-coding |
| 6281 | S100A10 | 1q21.3 | S100 calcium binding protein A10 | protein-coding |
| 6286 | S100P | 4p16.1 | S100 calcium binding protein P | protein-coding |
| 6300 | MAPK12 | 22q13.33 | mitogen-activated protein kinase 12 | protein-coding |
| 63035 | BCORL1 | Xq26.1 | BCL6 corepressor-like 1 | protein-coding |
| 6319 | SCD | 10q24.31 | stearoyl-CoA desaturase | protein-coding |
| 633 | BGN | Xq28 | biglycan | protein-coding |
| 6347 | CCL2 | 17q12 | C-C motif chemokine ligand 2 | protein-coding |
| 6348 | CCL3 | 17q12 | C-C motif chemokine ligand 3 | protein-coding |
| 6351 | CCL4 | 17q12 | C-C motif chemokine ligand 4 | protein-coding |
| 6352 | CCL5 | 17q12 | C-C motif chemokine ligand 5 | protein-coding |
| 6356 | CCL11 | 17q12 | C-C motif chemokine ligand 11 | protein-coding |
| 6357 | CCL13 | 17q12 | C-C motif chemokine ligand 13 | protein-coding |
| 6361 | CCL17 | 16q21 | C-C motif chemokine ligand 17 | protein-coding |
| 6362 | CCL18 | 17q12 | C-C motif chemokine ligand 18 | protein-coding |
| 6363 | CCL19 | 9p13.3 | C-C motif chemokine ligand 19 | protein-coding |
| 6366 | CCL21 | 9p13.3 | C-C motif chemokine ligand 21 | protein-coding |
| 6367 | CCL22 | 16q21 | C-C motif chemokine ligand 22 | protein-coding |
| 637 | BID | 22q11.21 | BH3 interacting domain death agonist | protein-coding |
| 6376 | CX3CL1 | 16q21 | C-X3-C motif chemokine ligand 1 | protein-coding |
| 6382 | SDC1 | 2p24.1 | syndecan 1 | protein-coding |
| 6387 | CXCL12 | 10q11.21 | C-X-C motif chemokine ligand 12 | protein-coding |
| 639 | PRDM1 | 6q21 | PR/SET domain 1 | protein-coding |
| 63917 | GALNT11 | 7q36.1|7q36.1 | polypeptide N-acetylgalactosaminyltransferase 11 | protein-coding |
| 63928 | CHP2 | 16p12.2 | calcineurin like EF-hand protein 2 | protein-coding |
| 63976 | PRDM16 | 1p36.32 | PR/SET domain 16 | protein-coding |
| 640 | BLK | 8p23.1 | BLK proto-oncogene, Src family tyrosine kinase | protein-coding |
| 6401 | SELE | 1q24.2 | selectin E | protein-coding |
| 6402 | SELL | 1q24.2 | selectin L | protein-coding |
| 6403 | SELP | 1q24.2 | selectin P | protein-coding |
| 6404 | SELPLG | 12q24.11 | selectin P ligand | protein-coding |
| 6406 | SEMG1 | 20q13.12 | semenogelin I | protein-coding |
| 641 | BLM | 15q26.1 | Bloom syndrome RecQ like helicase | protein-coding |
| 64109 | CRLF2 | Xp22.33 and Yp11.2 | cytokine receptor-like factor 2 | protein-coding |
| 64127 | NOD2 | 16q12.1 | nucleotide binding oligomerization domain containing 2 | protein-coding |
| 6416 | MAP2K4 | 17p12 | mitogen-activated protein kinase kinase 4 | protein-coding |
| 6418 | SET | 9q34.11 | SET nuclear proto-oncogene | protein-coding |
| 6419 | SETMAR | 3p26.1 | SET domain and mariner transposase fusion gene | protein-coding |
| 6422 | SFRP1 | 8p11.21 | secreted frizzled related protein 1 | protein-coding |
| 6423 | SFRP2 | 4q31.3 | secreted frizzled related protein 2 | protein-coding |
| 6425 | SFRP5 | 10q24.2 | secreted frizzled related protein 5 | protein-coding |
| 6426 | SRSF1 | 17q22 | serine and arginine rich splicing factor 1 | protein-coding |
| 6427 | SRSF2 | 17q25.1 | serine and arginine rich splicing factor 2 | protein-coding |
| 643 | CXCR5 | 11q23.3 | C-X-C motif chemokine receptor 5 | protein-coding |
| 64324 | NSD1 | 5q35.3 | nuclear receptor binding SET domain protein 1 | protein-coding |
| 644 | BLVRA | 7p13 | biliverdin reductase A | protein-coding |
| 6441 | SFTPD | 10q22.3 | surfactant protein D | protein-coding |
| 6452 | SH3BP2 | 4p16.3 | SH3 domain binding protein 2 | protein-coding |
| 6461 | SHB | 9p13.1 | SH2 domain containing adaptor protein B | protein-coding |
| 6464 | SHC1 | 1q21.3 | SHC adaptor protein 1 | protein-coding |
| 6469 | SHH | 7q36.3 | sonic hedgehog | protein-coding |
| 6470 | SHMT1 | 17p11.2 | serine hydroxymethyltransferase 1 | protein-coding |
| 64714 | PDIA2 | 16p13.3 | protein disulfide isomerase family A member 2 | protein-coding |
| 6476 | SI | 3q26.1 | sucrase-isomaltase | protein-coding |
| 6478 | SIAH2 | 3q25.1 | siah E3 ubiquitin protein ligase 2 | protein-coding |
| 64783 | RBM15 | 1p13.3 | RNA binding motif protein 15 | protein-coding |
| 648 | BMI1 | 10p12.2 | BMI1 proto-oncogene, polycomb ring finger | protein-coding |
| 6480 | ST6GAL1 | 3q27.3 | ST6 beta-galactoside alpha-2,6-sialyltransferase 1 | protein-coding |
| 64857 | PLEKHG2 | 19q13.2 | pleckstrin homology and RhoGEF domain containing G2 | protein-coding |
| 64919 | BCL11B | 14q32.2 | B-cell CLL/lymphoma 11B | protein-coding |
| 6497 | SKI | 1p36.33-p36.32 | SKI proto-oncogene | protein-coding |
| 6502 | SKP2 | 5p13.2 | S-phase kinase associated protein 2 | protein-coding |
| 65057 | ACD | 16q22.1 | ACD, shelterin complex subunit and telomerase recruitment factor | protein-coding |
| 6513 | SLC2A1 | 1p34.2 | solute carrier family 2 member 1 | protein-coding |
| 652 | BMP4 | 14q22.2 | bone morphogenetic protein 4 | protein-coding |
| 6530 | SLC6A2 | 16q12.2 | solute carrier family 6 member 2 | protein-coding |
| 653220 | XAGE1B | Xp11.22 | X antigen family member 1B | protein-coding |
| 654 | BMP6 | 6p24.3 | bone morphogenetic protein 6 | protein-coding |
| 6548 | SLC9A1 | 1p36.11 | solute carrier family 9 member A1 | protein-coding |
| 6573 | SLC19A1 | 21q22.3 | solute carrier family 19 member 1 | protein-coding |
| 6580 | SLC22A1 | 6q25.3 | solute carrier family 22 member 1 | protein-coding |
| 6597 | SMARCA4 | 19p13.2 | SWI/SNF related, matrix associated, actin dependent regulator of chromatin, subfamily a, member 4 | protein-coding |
| 6598 | SMARCB1 | 22q11.23|22q11 | SWI/SNF related, matrix associated, actin dependent regulator of chromatin, subfamily b, member 1 | protein-coding |
| 6608 | SMO | 7q32.1 | smoothened, frizzled class receptor | protein-coding |
| 6610 | SMPD2 | 6q21 | sphingomyelin phosphodiesterase 2 | protein-coding |
| 6612 | SUMO3 | 21q22.3 | small ubiquitin-like modifier 3 | protein-coding |
| 6613 | SUMO2 | 17q25.1 | small ubiquitin-like modifier 2 | protein-coding |
| 6624 | FSCN1 | 7p22.1 | fascin actin-bundling protein 1 | protein-coding |
| 6638 | SNRPN | 15q11.2 | small nuclear ribonucleoprotein polypeptide N | protein-coding |
| 664 | BNIP3 | 10q26.3 | BCL2 interacting protein 3 | protein-coding |
| 6646 | SOAT1 | 1q25.2 | sterol O-acyltransferase 1 | protein-coding |
| 6647 | SOD1 | 21q22.11 | superoxide dismutase 1, soluble | protein-coding |
| 6648 | SOD2 | 6q25.3 | superoxide dismutase 2, mitochondrial | protein-coding |
| 6653 | SORL1 | 11q24.1 | sortilin related receptor 1 | protein-coding |
| 6654 | SOS1 | 2p22.1 | SOS Ras/Rac guanine nucleotide exchange factor 1 | protein-coding |
| 6659 | SOX4 | 6p22.3 | SRY-box 4 | protein-coding |
| 6660 | SOX5 | 12p12.1 | SRY-box 5 | protein-coding |
| 6664 | SOX11 | 2p25.2 | SRY-box 11 | protein-coding |
| 6667 | SP1 | 12q13.13 | Sp1 transcription factor | protein-coding |
| 6672 | SP100 | 2q37.1 | SP100 nuclear antigen | protein-coding |
| 6678 | SPARC | 5q33.1 | secreted protein acidic and cysteine rich | protein-coding |
| 668 | FOXL2 | 3q22.3 | forkhead box L2 | protein-coding |
| 6688 | SPI1 | 11p11.2 | Spi-1 proto-oncogene | protein-coding |
| 6691 | SPINK2 | 4q12 | serine peptidase inhibitor, Kazal type 2 | protein-coding |
| 6696 | SPP1 | 4q22.1 | secreted phosphoprotein 1 | protein-coding |
| 6714 | SRC | 20q11.23 | SRC proto-oncogene, non-receptor tyrosine kinase | protein-coding |
| 6717 | SRI | 7q21.12 | sorcin | protein-coding |
| 672 | BRCA1 | 17q21.31 | BRCA1, DNA repair associated | protein-coding |
| 673 | BRAF | 7q34 | B-Raf proto-oncogene, serine/threonine kinase | protein-coding |
| 6733 | SRPK2 | 7q22.3 | SRSF protein kinase 2 | protein-coding |
| 675 | BRCA2 | 13q13.1 | BRCA2, DNA repair associated | protein-coding |
| 6761 | ST2 | 11p14.3-p12 | suppression of tumorigenicity 2 | other |
| 677 | ZFP36L1 | 14q24.1 | ZFP36 ring finger protein like 1 | protein-coding |
| 6772 | STAT1 | 2q32.2 | signal transducer and activator of transcription 1 | protein-coding |
| 6774 | STAT3 | 17q21.2 | signal transducer and activator of transcription 3 | protein-coding |
| 6776 | STAT5A | 17q21.2 | signal transducer and activator of transcription 5A | protein-coding |
| 6777 | STAT5B | 17q21.2 | signal transducer and activator of transcription 5B | protein-coding |
| 6778 | STAT6 | 12q13.3 | signal transducer and activator of transcription 6 | protein-coding |
| 678 | ZFP36L2 | 2p21 | ZFP36 ring finger protein like 2 | protein-coding |
| 6781 | STC1 | 8p21.2 | stanniocalcin 1 | protein-coding |
| 6789 | STK4 | 20q13.12 | serine/threonine kinase 4 | protein-coding |
| 6790 | AURKA | 20q13.2 | aurora kinase A | protein-coding |
| 6794 | STK11 | 19p13.3 | serine/threonine kinase 11 | protein-coding |
| 6795 | AURKC | 19q13.43 | aurora kinase C | protein-coding |
| 682 | BSG | 19p13.3 | basigin (Ok blood group) | protein-coding |
| 6850 | SYK | 9q22.2 | spleen associated tyrosine kinase | protein-coding |
| 6863 | TAC1 | 7q21.3 | tachykinin precursor 1 | protein-coding |
| 6868 | ADAM17 | 2p25.1 | ADAM metallopeptidase domain 17 | protein-coding |
| 6869 | TACR1 | 2p12 | tachykinin receptor 1 | protein-coding |
| 688 | KLF5 | 13q22.1 | Kruppel like factor 5 | protein-coding |
| 6886 | TAL1 | 1p33 | TAL bHLH transcription factor 1, erythroid differentiation factor | protein-coding |
| 6890 | TAP1 | 6p21.32 | transporter 1, ATP binding cassette subfamily B member | protein-coding |
| 6891 | TAP2 | 6p21.32 | transporter 2, ATP binding cassette subfamily B member | protein-coding |
| 6925 | TCF4 | 18q21.2 | transcription factor 4 | protein-coding |
| 6929 | TCF3 | 19p13.3 | transcription factor 3 | protein-coding |
| 693124 | MIR532 | Xp11.23 | microRNA 532 | ncRNA |
| 693149 | MIR564 | 3p21.31 | microRNA 564 | ncRNA |
| 693197 | MIR612 | 11q13.1 | microRNA 612 | ncRNA |
| 693218 | MIR633 | 17q23.2 | microRNA 633 | ncRNA |
| 693223 | MIR638 | 19p13.2 | microRNA 638 | ncRNA |
| 6934 | TCF7L2 | 10q25.2-q25.3 | transcription factor 7 like 2 | protein-coding |
| 6935 | ZEB1 | 10p11.22 | zinc finger E-box binding homeobox 1 | protein-coding |
| 6938 | TCF12 | 15q21.3 | transcription factor 12 | protein-coding |
| 694 | BTG1 | 12q21.33 | BTG anti-proliferation factor 1 | protein-coding |
| 695 | BTK | Xq22.1 | Bruton tyrosine kinase | protein-coding |
| 6955 | TRA | 14q11.2 | T-cell receptor alpha locus | protein-coding |
| 6957 | TRB | 7q34 | T cell receptor beta locus | protein-coding |
| 6964 | TRD | 14q11.2 | T cell receptor delta locus | protein-coding |
| 6965 | TRG | 7p14.1 | T cell receptor gamma locus | protein-coding |
| 699 | BUB1 | 2q13 | BUB1 mitotic checkpoint serine/threonine kinase | protein-coding |
| 6996 | TDG | 12q23.3 | thymine DNA glycosylase | protein-coding |
| 7001 | PRDX2 | 19p13.13 | peroxiredoxin 2 | protein-coding |
| 701 | BUB1B | 15q15.1 | BUB1 mitotic checkpoint serine/threonine kinase B | protein-coding |
| 7010 | TEK | 9p21.2 | TEK receptor tyrosine kinase | protein-coding |
| 7012 | TERC | 3q26.2 | telomerase RNA component | ncRNA |
| 7013 | TERF1 | 8q21.11 | telomeric repeat binding factor 1 | protein-coding |
| 7014 | TERF2 | 16q22.1 | telomeric repeat binding factor 2 | protein-coding |
| 7015 | TERT | 5p15.33 | telomerase reverse transcriptase | protein-coding |
| 7036 | TFR2 | 7q22.1 | transferrin receptor 2 | protein-coding |
| 7037 | TFRC | 3q29 | transferrin receptor | protein-coding |
| 7040 | TGFB1 | 19q13.2 | transforming growth factor beta 1 | protein-coding |
| 7046 | TGFBR1 | 9q22.33 | transforming growth factor beta receptor 1 | protein-coding |
| 7050 | TGIF1 | 18p11.31 | TGFB induced factor homeobox 1 | protein-coding |
| 7052 | TGM2 | 20q11.23 | transglutaminase 2 | protein-coding |
| 7056 | THBD | 20p11.21 | thrombomodulin | protein-coding |
| 7057 | THBS1 | 15q14 | thrombospondin 1 | protein-coding |
| 7066 | THPO | 3q27.1 | thrombopoietin | protein-coding |
| 7068 | THRB | 3p24.2 | thyroid hormone receptor beta | protein-coding |
| 7070 | THY1 | 11q23.3 | Thy-1 cell surface antigen | protein-coding |
| 7071 | KLF10 | 8q22.3 | Kruppel like factor 10 | protein-coding |
| 7073 | TIAL1 | 10q26.11 | TIA1 cytotoxic granule associated RNA binding protein like 1 | protein-coding |
| 7074 | TIAM1 | 21q22.11 | T-cell lymphoma invasion and metastasis 1 | protein-coding |
| 7076 | TIMP1 | Xp11.3 | TIMP metallopeptidase inhibitor 1 | protein-coding |
| 7077 | TIMP2 | 17q25.3 | TIMP metallopeptidase inhibitor 2 | protein-coding |
| 7078 | TIMP3 | 22q12.3 | TIMP metallopeptidase inhibitor 3 | protein-coding |
| 7080 | NKX2-1 | 14q13.3 | NK2 homeobox 1 | protein-coding |
| 7082 | TJP1 | 15q13.1 | tight junction protein 1 | protein-coding |
| 7097 | TLR2 | 4q31.3 | toll like receptor 2 | protein-coding |
| 7098 | TLR3 | 4q35.1 | toll like receptor 3 | protein-coding |
| 7099 | TLR4 | 9q33.1 | toll like receptor 4 | protein-coding |
| 7112 | TMPO | 12q23.1 | thymopoietin | protein-coding |
| 7124 | TNF | 6p21.33 | tumor necrosis factor | protein-coding |
| 7128 | TNFAIP3 | 6q23.3 | TNF alpha induced protein 3 | protein-coding |
| 7132 | TNFRSF1A | 12p13.31 | TNF receptor superfamily member 1A | protein-coding |
| 714 | C1QC | 1p36.12 | complement C1q C chain | protein-coding |
| 7150 | TOP1 | 20q12 | topoisomerase (DNA) I | protein-coding |
| 7153 | TOP2A | 17q21.2 | topoisomerase (DNA) II alpha | protein-coding |
| 7155 | TOP2B | 3p24.2 | topoisomerase (DNA) II beta | protein-coding |
| 7156 | TOP3A | 17p11.2 | topoisomerase (DNA) III alpha | protein-coding |
| 7157 | TP53 | 17p13.1 | tumor protein p53 | protein-coding |
| 7159 | TP53BP2 | 1q41 | tumor protein p53 binding protein 2 | protein-coding |
| 7161 | TP73 | 1p36.32 | tumor protein p73 | protein-coding |
| 7163 | TPD52 | 8q21.13 | tumor protein D52 | protein-coding |
| 7170 | TPM3 | 1q21.3 | tropomyosin 3 | protein-coding |
| 7172 | TPMT | 6p22.3 | thiopurine S-methyltransferase | protein-coding |
| 7173 | TPO | 2p25.3 | thyroid peroxidase | protein-coding |
| 7181 | NR2C1 | 12q22 | nuclear receptor subfamily 2 group C member 1 | protein-coding |
| 7186 | TRAF2 | 9q34.3 | TNF receptor associated factor 2 | protein-coding |
| 7187 | TRAF3 | 14q32.32 | TNF receptor associated factor 3 | protein-coding |
| 723778 | MIR650 | 22q11.22 | microRNA 650 | ncRNA |
| 724033 | MIR663A | 20p11.1 | microRNA 663a | ncRNA |
| 7249 | TSC2 | 16p13.3 | tuberous sclerosis 2 | protein-coding |
| 726 | CAPN5 | 11q13.5 | calpain 5 | protein-coding |
| 728911 | CT45A2 | Xq26.3 | cancer/testis antigen family 45 member A2 | protein-coding |
| 7291 | TWIST1 | 7p21.1 | twist family bHLH transcription factor 1 | protein-coding |
| 7297 | TYK2 | 19p13.2 | tyrosine kinase 2 | protein-coding |
| 7298 | TYMS | 18p11.32 | thymidylate synthetase | protein-coding |
| 7305 | TYROBP | 19q13.12 | TYRO protein tyrosine kinase binding protein | protein-coding |
| 7307 | U2AF1 | 21q22.3 | U2 small nuclear RNA auxiliary factor 1 | protein-coding |
| 7318 | UBA7 | 3p21.31 | ubiquitin like modifier activating enzyme 7 | protein-coding |
| 7337 | UBE3A | 15q11.2 | ubiquitin protein ligase E3A | protein-coding |
| 7341 | SUMO1 | 2q33.1 | small ubiquitin-like modifier 1 | protein-coding |
| 7357 | UGCG | 9q31.3 | UDP-glucose ceramide glucosyltransferase | protein-coding |
| 7367 | UGT2B17 | 4q13.2 | UDP glucuronosyltransferase family 2 member B17 | protein-coding |
| 7392 | USF2 | 19q13.12 | upstream transcription factor 2, c-fos interacting | protein-coding |
| 7403 | KDM6A | Xp11.3 | lysine demethylase 6A | protein-coding |
| 7409 | VAV1 | 19p13.3 | vav guanine nucleotide exchange factor 1 | protein-coding |
| 7412 | VCAM1 | 1p21.2 | vascular cell adhesion molecule 1 | protein-coding |
| 7415 | VCP | 9p13.3 | valosin containing protein | protein-coding |
| 7421 | VDR | 12q13.11 | vitamin D (1,25- dihydroxyvitamin D3) receptor | protein-coding |
| 7422 | VEGFA | 6p21.1 | vascular endothelial growth factor A | protein-coding |
| 7424 | VEGFC | 4q34.3 | vascular endothelial growth factor C | protein-coding |
| 7430 | EZR | 6q25.3 | ezrin | protein-coding |
| 7450 | VWF | 12p13.31 | von Willebrand factor | protein-coding |
| 7462 | LAT2 | 7q11.23 | linker for activation of T-cells family member 2 | protein-coding |
| 7465 | WEE1 | 11p15.4 | WEE1 G2 checkpoint kinase | protein-coding |
| 7468 | NSD2 | 4p16.3 | nuclear receptor binding SET domain protein 2 | protein-coding |
| 7473 | WNT3 | 17q21.31-q21.32 | Wnt family member 3 | protein-coding |
| 7474 | WNT5A | 3p14.3 | Wnt family member 5A | protein-coding |
| 7477 | WNT7B | 22q13.31 | Wnt family member 7B | protein-coding |
| 7483 | WNT9A | 1q42.13 | Wnt family member 9A | protein-coding |
| 7486 | WRN | 8p12 | Werner syndrome RecQ like helicase | protein-coding |
| 7490 | WT1 | 11p13 | Wilms tumor 1 | protein-coding |
| 7494 | XBP1 | 22q12.1|22q12 | X-box binding protein 1 | protein-coding |
| 7507 | XPA | 9q22.33 | XPA, DNA damage recognition and repair factor | protein-coding |
| 7508 | XPC | 3p25.1 | XPC complex subunit, DNA damage recognition and repair factor | protein-coding |
| 7514 | XPO1 | 2p15 | exportin 1 | protein-coding |
| 7515 | XRCC1 | 19q13.31 | X-ray repair cross complementing 1 | protein-coding |
| 7517 | XRCC3 | 14q32.33 | X-ray repair cross complementing 3 | protein-coding |
| 7518 | XRCC4 | 5q14.2 | X-ray repair cross complementing 4 | protein-coding |
| 752 | FMNL1 | 17q21.31 | formin like 1 | protein-coding |
| 7520 | XRCC5 | 2q35 | X-ray repair cross complementing 5 | protein-coding |
| 7528 | YY1 | 14q32.2 | YY1 transcription factor | protein-coding |
| 7533 | YWHAH | 22q12.3 | tyrosine 3-monooxygenase/tryptophan 5-monooxygenase activation protein eta | protein-coding |
| 7535 | ZAP70 | 2q11.2 | zeta chain of T cell receptor associated protein kinase 70 | protein-coding |
| 7543 | ZFX | Xp22.11 | zinc finger protein, X-linked | protein-coding |
| 7704 | ZBTB16 | 11q23.2 | zinc finger and BTB domain containing 16 | protein-coding |
| 771 | CA12 | 15q22.2 | carbonic anhydrase 12 | protein-coding |
| 7750 | ZMYM2 | 13q12.11 | zinc finger MYM-type containing 2 | protein-coding |
| 7799 | PRDM2 | 1p36.21 | PR/SET domain 2 | protein-coding |
| 780 | DDR1 | 6p21.33 | discoidin domain receptor tyrosine kinase 1 | protein-coding |
| 7839 | LEPQTL1 | 2p21 | Leptin, serum levels of | unknown |
| 7852 | CXCR4 | 2q22.1 | C-X-C motif chemokine receptor 4 | protein-coding |
| 7862 | BRPF1 | 3p25.3 | bromodomain and PHD finger containing 1 | protein-coding |
| 79017 | GGCT | 7p14.3 | gamma-glutamylcyclotransferase | protein-coding |
| 7903 | ST8SIA4 | 5q21.1 | ST8 alpha-N-acetyl-neuraminide alpha-2,8-sialyltransferase 4 | protein-coding |
| 79054 | TRPM8 | 2q37.1 | transient receptor potential cation channel subfamily M member 8 | protein-coding |
| 7913 | DEK | 6p22.3 | DEK proto-oncogene | protein-coding |
| 79155 | TNIP2 | 4p16.3 | TNFAIP3 interacting protein 2 | protein-coding |
| 79365 | BHLHE41 | 12p12.1 | basic helix-loop-helix family member e41 | protein-coding |
| 79368 | FCRL2 | 1q23.1 | Fc receptor like 2 | protein-coding |
| 79370 | BCL2L14 | 12p13.2 | BCL2 like 14 | protein-coding |
| 79444 | BIRC7 | 20q13.33 | baculoviral IAP repeat containing 7 | protein-coding |
| 79465 | ULBP3 | 6q25.1 | UL16 binding protein 3 | protein-coding |
| 796 | CALCA | 11p15.2 | calcitonin related polypeptide alpha | protein-coding |
| 79602 | ADIPOR2 | 12p13.33 | adiponectin receptor 2 | protein-coding |
| 79648 | MCPH1 | 8p23.1 | microcephalin 1 | protein-coding |
| 79682 | CENPU | 4q35.1 | centromere protein U | protein-coding |
| 79718 | TBL1XR1 | 3q26.32 | transducin beta like 1 X-linked receptor 1 | protein-coding |
| 79727 | LIN28A | 1p36.11 | lin-28 homolog A | protein-coding |
| 7980 | TFPI2 | 7q21.3 | tissue factor pathway inhibitor 2 | protein-coding |
| 79831 | KDM8 | 16p12.1 | lysine demethylase 8 | protein-coding |
| 79870 | BAALC | 8q22.3 | brain and acute leukemia, cytoplasmic | protein-coding |
| 7994 | KAT6A | 8p11.21 | lysine acetyltransferase 6A | protein-coding |
| 79971 | WLS | 1p31.3 | wntless Wnt ligand secretion mediator | protein-coding |
| 80010 | RMI1 | 9q21.32 | RecQ mediated genome instability 1 | protein-coding |
| 8013 | NR4A3 | 9q31.1 | nuclear receptor subfamily 4 group A member 3 | protein-coding |
| 8019 | BRD3 | 9q34.2 | bromodomain containing 3 | protein-coding |
| 80206 | FHOD3 | 18q12.2 | formin homology 2 domain containing 3 | protein-coding |
| 8021 | NUP214 | 9q34.13 | nucleoporin 214 | protein-coding |
| 80237 | ELL3 | 15q15.3 | elongation factor for RNA polymerase II 3 | protein-coding |
| 8028 | MLLT10 | 10p12.31 | myeloid/lymphoid or mixed-lineage leukemia; translocated to, 10 | protein-coding |
| 80309 | SPHKAP | 2q36.3 | SPHK1 interactor, AKAP domain containing | protein-coding |
| 80312 | TET1 | 10q21.3 | tet methylcytosine dioxygenase 1 | protein-coding |
| 80314 | EPC1 | 10p11.22 | enhancer of polycomb homolog 1 | protein-coding |
| 80326 | WNT10A | 2q35 | Wnt family member 10A | protein-coding |
| 80328 | ULBP2 | 6q25.1 | UL16 binding protein 2 | protein-coding |
| 80329 | ULBP1 | 6q25.1 | UL16 binding protein 1 | protein-coding |
| 80380 | PDCD1LG2 | 9p24.1 | programmed cell death 1 ligand 2 | protein-coding |
| 80705 | TSGA10 | 2q11.2 | testis specific 10 | protein-coding |
| 80781 | COL18A1 | 21q22.3 | collagen type XVIII alpha 1 chain | protein-coding |
| 80824 | DUSP16 | 12p13.2 | dual specificity phosphatase 16 | protein-coding |
| 8085 | KMT2D | 12q13.12 | lysine methyltransferase 2D | protein-coding |
| 8091 | HMGA2 | 12q14.3 | high mobility group AT-hook 2 | protein-coding |
| 81029 | WNT5B | 12p13.33 | Wnt family member 5B | protein-coding |
| 811 | CALR | 19p13.13 | calreticulin | protein-coding |
| 8115 | TCL1A | 14q32.13 | T-cell leukemia/lymphoma 1A | protein-coding |
| 8148 | TAF15 | 17q12 | TATA-box binding protein associated factor 15 | protein-coding |
| 81608 | FIP1L1 | 4q12 | factor interacting with PAPOLA and CPSF1 | protein-coding |
| 8161 | COIL | 17q22 | coilin | protein-coding |
| 81617 | CAB39L | 13q14.2 | calcium binding protein 39 like | protein-coding |
| 81620 | CDT1 | 16q24.3 | chromatin licensing and DNA replication factor 1 | protein-coding |
| 81631 | MAP1LC3B | 16q24.2 | microtubule associated protein 1 light chain 3 beta | protein-coding |
| 8178 | ELL | 19p13.11 | elongation factor for RNA polymerase II | protein-coding |
| 818 | CAMK2G | 10q22.2 | calcium/calmodulin dependent protein kinase II gamma | protein-coding |
| 81848 | SPRY4 | 5q31.3 | sprouty RTK signaling antagonist 4 | protein-coding |
| 819 | CAMLG | 5q31.1 | calcium modulating ligand | protein-coding |
| 820 | CAMP | 3p21.31 | cathelicidin antimicrobial peptide | protein-coding |
| 8239 | USP9X | Xp11.4 | ubiquitin specific peptidase 9, X-linked | protein-coding |
| 824 | CAPN2 | 1q41 | calpain 2 | protein-coding |
| 8243 | SMC1A | Xp11.22 | structural maintenance of chromosomes 1A | protein-coding |
| 8277 | TKTL1 | Xq28 | transketolase like 1 | protein-coding |
| 8301 | PICALM | 11q14.2 | phosphatidylinositol binding clathrin assembly protein | protein-coding |
| 8312 | AXIN1 | 16p13.3 | axin 1 | protein-coding |
| 8322 | FZD4 | 11q14.2 | frizzled class receptor 4 | protein-coding |
| 8323 | FZD6 | 8q22.3 | frizzled class receptor 6 | protein-coding |
| 8326 | FZD9 | 7q11.23 | frizzled class receptor 9 | protein-coding |
| 8328 | GFI1B | 9q34.13 | growth factor independent 1B transcriptional repressor | protein-coding |
| 83416 | FCRL5 | 1q23.1 | Fc receptor like 5 | protein-coding |
| 83417 | FCRL4 | 1q23.1 | Fc receptor like 4 | protein-coding |
| 83439 | TCF7L1 | 2p11.2 | transcription factor 7 like 1 | protein-coding |
| 835 | CASP2 | 7q34 | caspase 2 | protein-coding |
| 83595 | SOX7 | 8p23.1 | SRY-box 7 | protein-coding |
| 83596 | BCL2L12 | 19q13.33 | BCL2 like 12 | protein-coding |
| 836 | CASP3 | 4q35.1 | caspase 3 | protein-coding |
| 83639 | TEX101 | 19q13.31 | testis expressed 101 | protein-coding |
| 83737 | ITCH | 20q11.22 | itchy E3 ubiquitin protein ligase | protein-coding |
| 83881 | MIXL1 | 1q42.12 | Mix paired-like homeobox | protein-coding |
| 83937 | RASSF4 | 10q11.21 | Ras association domain family member 4 | protein-coding |
| 83939 | EIF2A | 3q25.1 | eukaryotic translation initiation factor 2A | protein-coding |
| 840 | CASP7 | 10q25.3 | caspase 7 | protein-coding |
| 84002 | B3GNT5 | 3q27.1 | UDP-GlcNAc:betaGal beta-1,3-N-acetylglucosaminyltransferase 5 | protein-coding |
| 8405 | SPOP | 17q21.33 | speckle type BTB/POZ protein | protein-coding |
| 84081 | NSRP1 | 17q11.2 | nuclear speckle splicing regulatory protein 1 | protein-coding |
| 841 | CASP8 | 2q33.1 | caspase 8 | protein-coding |
| 84101 | USP44 | 12q22 | ubiquitin specific peptidase 44 | protein-coding |
| 84106 | PRAM1 | 19p13.2 | PML-RARA regulated adaptor molecule 1 | protein-coding |
| 84132 | USP42 | 7p22.1 | ubiquitin specific peptidase 42 | protein-coding |
| 84159 | ARID5B | 10q21.2 | AT-rich interaction domain 5B | protein-coding |
| 842 | CASP9 | 1p36.21 | caspase 9 | protein-coding |
| 84289 | ING5 | 2q37.3 | inhibitor of growth family member 5 | protein-coding |
| 84295 | PHF6 | Xq26.2 | PHD finger protein 6 | protein-coding |
| 843 | CASP10 | 2q33.1 | caspase 10 | protein-coding |
| 84432 | PROK1 | 1p13.3 | prokineticin 1 | protein-coding |
| 84433 | CARD11 | 7p22.2 | caspase recruitment domain family member 11 | protein-coding |
| 84444 | DOT1L | 19p13.3 | DOT1 like histone lysine methyltransferase | protein-coding |
| 84528 | RHOXF2 | Xq24 | Rhox homeobox family member 2 | protein-coding |
| 84557 | MAP1LC3A | 20q11.22 | microtubule associated protein 1 light chain 3 alpha | protein-coding |
| 84662 | GLIS2 | 16p13.3 | GLIS family zinc finger 2 | protein-coding |
| 8467 | SMARCA5 | 4q31.21 | SWI/SNF related, matrix associated, actin dependent regulator of chromatin, subfamily a, member 5 | protein-coding |
| 84678 | KDM2B | 12q24.31 | lysine demethylase 2B | protein-coding |
| 847 | CAT | 11p13 | catalase | protein-coding |
| 84707 | BEX2 | Xq22.2 | brain expressed X-linked 2 | protein-coding |
| 84868 | HAVCR2 | 5q33.3 | hepatitis A virus cellular receptor 2 | protein-coding |
| 84883 | AIFM2 | 10q22.1 | apoptosis inducing factor, mitochondria associated 2 | protein-coding |
| 84911 | ZNF382 | 19q13.12 | zinc finger protein 382 | protein-coding |
| 84922 | FIZ1 | 19q13.42 | FLT3 interacting zinc finger 1 | protein-coding |
| 84939 | MUM1 | 19p13.3 | melanoma associated antigen (mutated) 1 | protein-coding |
| 84959 | UBASH3B | 11q24.1 | ubiquitin associated and SH3 domain containing B | protein-coding |
| 8498 | RANBP3 | 19p13.3 | RAN binding protein 3 | protein-coding |
| 85021 | REPS1 | 6q24.1 | RALBP1 associated Eps domain containing 1 | protein-coding |
| 8517 | IKBKG | Xq28 | inhibitor of kappa light polypeptide gene enhancer in B-cells, kinase gamma | protein-coding |
| 85320 | ABCC11 | 16q12.1 | ATP binding cassette subfamily C member 11 | protein-coding |
| 85413 | SLC22A16 | 6q21|6q21-q22.1 | solute carrier family 22 member 16 | protein-coding |
| 8544 | PIR | Xp22.2 | pirin | protein-coding |
| 8553 | BHLHE40 | 3p26.1 | basic helix-loop-helix family member e40 | protein-coding |
| 8569 | MKNK1 | 1p33 | MAP kinase interacting serine/threonine kinase 1 | protein-coding |
| 857 | CAV1 | 7q31.2 | caveolin 1 | protein-coding |
| 860 | RUNX2 | 6p21.1 | runt related transcription factor 2 | protein-coding |
| 8600 | TNFSF11 | 13q14.11 | tumor necrosis factor superfamily member 11 | protein-coding |
| 8607 | RUVBL1 | 3q21.3 | RuvB like AAA ATPase 1 | protein-coding |
| 861 | RUNX1 | 21q22.12 | runt related transcription factor 1 | protein-coding |
| 862 | RUNX1T1 | 8q21.3 | RUNX1 translocation partner 1 | protein-coding |
| 8626 | TP63 | 3q28 | tumor protein p63 | protein-coding |
| 863 | CBFA2T3 | 16q24.3 | CBFA2/RUNX1 translocation partner 3 | protein-coding |
| 864 | RUNX3 | 1p36.11 | runt related transcription factor 3 | protein-coding |
| 8644 | AKR1C3 | 10p15.1 | aldo-keto reductase family 1 member C3 | protein-coding |
| 865 | CBFB | 16q22.1 | core-binding factor beta subunit | protein-coding |
| 8650 | NUMB | 14q24.2-q24.3 | NUMB, endocytic adaptor protein | protein-coding |
| 8651 | SOCS1 | 16p13.13 | suppressor of cytokine signaling 1 | protein-coding |
| 867 | CBL | 11q23.3 | Cbl proto-oncogene | protein-coding |
| 8678 | BECN1 | 17q21.31 | beclin 1 | protein-coding |
| 868 | CBLB | 3q13.11 | Cbl proto-oncogene B | protein-coding |
| 8714 | ABCC3 | 17q21.33 | ATP binding cassette subfamily C member 3 | protein-coding |
| 8726 | EED | 11q14.2 | embryonic ectoderm development | protein-coding |
| 873 | CBR1 | 21q22.12 | carbonyl reductase 1 | protein-coding |
| 8737 | RIPK1 | 6p25.2 | receptor interacting serine/threonine kinase 1 | protein-coding |
| 8739 | HRK | 12q24.22 | harakiri, BCL2 interacting protein | protein-coding |
| 8741 | TNFSF13 | 17p13.1 | tumor necrosis factor superfamily member 13 | protein-coding |
| 8743 | TNFSF10 | 3q26.31 | tumor necrosis factor superfamily member 10 | protein-coding |
| 8744 | TNFSF9 | 19p13.3 | tumor necrosis factor superfamily member 9 | protein-coding |
| 875 | CBS | 21q22.3 | cystathionine-beta-synthase | protein-coding |
| 8778 | SIGLEC5 | 19q13.41 | sialic acid binding Ig like lectin 5 | protein-coding |
| 8788 | DLK1 | 14q32.2 | delta like non-canonical Notch ligand 1 | protein-coding |
| 8792 | TNFRSF11A | 18q21.33 | TNF receptor superfamily member 11a | protein-coding |
| 8793 | TNFRSF10D | 8p21.3 | TNF receptor superfamily member 10d | protein-coding |
| 8794 | TNFRSF10C | 8p21.3 | TNF receptor superfamily member 10c | protein-coding |
| 8795 | TNFRSF10B | 8p21.3 | TNF receptor superfamily member 10b | protein-coding |
| 8797 | TNFRSF10A | 8p21.3 | TNF receptor superfamily member 10a | protein-coding |
| 8805 | TRIM24 | 7q33-q34 | tripartite motif containing 24 | protein-coding |
| 8821 | INPP4B | 4q31.21 | inositol polyphosphate-4-phosphatase type II B | protein-coding |
| 8828 | NRP2 | 2q33.3 | neuropilin 2 | protein-coding |
| 8829 | NRP1 | 10p11.22 | neuropilin 1 | protein-coding |
| 8832 | CD84 | 1q23.3 | CD84 molecule | protein-coding |
| 8835 | SOCS2 | 12q22 | suppressor of cytokine signaling 2 | protein-coding |
| 8836 | GGH | 8q12.3 | gamma-glutamyl hydrolase | protein-coding |
| 8837 | CFLAR | 2q33.1 | CASP8 and FADD like apoptosis regulator | protein-coding |
| 8841 | HDAC3 | 5q31.3 | histone deacetylase 3 | protein-coding |
| 8842 | PROM1 | 4p15.32 | prominin 1 | protein-coding |
| 8844 | KSR1 | 17q11.2 | kinase suppressor of ras 1 | protein-coding |
| 8847 | DLEU2 | 13q14.2 | deleted in lymphocytic leukemia 2 (non-protein coding) | ncRNA |
| 8848 | TSC22D1 | 13q14.11 | TSC22 domain family member 1 | protein-coding |
| 8861 | LDB1 | 10q24.32 | LIM domain binding 1 | protein-coding |
| 8863 | PER3 | 1p36.23 | period circadian clock 3 | protein-coding |
| 8864 | PER2 | 2q37.3 | period circadian clock 2 | protein-coding |
| 8869 | ST3GAL5 | 2p11.2 | ST3 beta-galactoside alpha-2,3-sialyltransferase 5 | protein-coding |
| 887 | CCKBR | 11p15.4 | cholecystokinin B receptor | protein-coding |
| 8870 | IER3 | 6p21.33 | immediate early response 3 | protein-coding |
| 8871 | SYNJ2 | 6q25.3 | synaptojanin 2 | protein-coding |
| 8877 | SPHK1 | 17q25.1 | sphingosine kinase 1 | protein-coding |
| 8878 | SQSTM1 | 5q35.3 | sequestosome 1 | protein-coding |
| 8886 | DDX18 | 2q14.1 | DEAD-box helicase 18 | protein-coding |
| 8887 | TAX1BP1 | 7p15.2 | Tax1 binding protein 1 | protein-coding |
| 890 | CCNA2 | 4q27 | cyclin A2 | protein-coding |
| 8900 | CCNA1 | 13q13.3 | cyclin A1 | protein-coding |
| 891 | CCNB1 | 5q13.2 | cyclin B1 | protein-coding |
| 8915 | BCL10 | 1p22.3 | B-cell CLL/lymphoma 10 | protein-coding |
| 8936 | WASF1 | 6q21 | WAS protein family member 1 | protein-coding |
| 894 | CCND2 | 12p13.32 | cyclin D2 | protein-coding |
| 896 | CCND3 | 6p21.1 | cyclin D3 | protein-coding |
| 898 | CCNE1 | 19q12 | cyclin E1 | protein-coding |
| 8996 | NOL3 | 16q22.1 | nucleolar protein 3 | protein-coding |
| 9 | NAT1 | 8p22 | N-acetyltransferase 1 | protein-coding |
| 900 | CCNG1 | 5q34 | cyclin G1 | protein-coding |
| 9020 | MAP3K14 | 17q21.31 | mitogen-activated protein kinase kinase kinase 14 | protein-coding |
| 9021 | SOCS3 | 17q25.3 | suppressor of cytokine signaling 3 | protein-coding |
| 9043 | SPAG9 | 17q21.33 | sperm associated antigen 9 | protein-coding |
| 9046 | DOK2 | 8p21.3 | docking protein 2 | protein-coding |
| 90480 | GADD45GIP1 | 19p13.13 | GADD45G interacting protein 1 | protein-coding |
| 9057 | SLC7A6 | 16q22.1 | solute carrier family 7 member 6 | protein-coding |
| 90865 | IL33 | 9p24.1 | interleukin 33 | protein-coding |
| 91039 | DPP9 | 19p13.3 | dipeptidyl peptidase 9 | protein-coding |
| 911 | CD1C | 1q23.1 | CD1c molecule | protein-coding |
| 912 | CD1D | 1q23.1 | CD1d molecule | protein-coding |
| 9126 | SMC3 | 10q25.2 | structural maintenance of chromosomes 3 | protein-coding |
| 9139 | CBFA2T2 | 20q11.21-q11.22 | CBFA2/RUNX1 translocation partner 2 | protein-coding |
| 914 | CD2 | 1p13.1 | CD2 molecule | protein-coding |
| 9141 | PDCD5 | 19q13.11 | programmed cell death 5 | protein-coding |
| 9154 | SLC28A1 | 15q25.3 | solute carrier family 28 member 1 | protein-coding |
| 9166 | EBAG9 | 8q23.2 | estrogen receptor binding site associated, antigen, 9 | protein-coding |
| 917 | CD3G | 11q23.3 | CD3g molecule | protein-coding |
| 9170 | LPAR2 | 19p13.11 | lysophosphatidic acid receptor 2 | protein-coding |
| 91875 | TTC5 | 14q11.2 | tetratricopeptide repeat domain 5 | protein-coding |
| 919 | CD247 | 1q24.2 | CD247 molecule | protein-coding |
| 91975 | ZNF300 | 5q33.1 | zinc finger protein 300 | protein-coding |
| 920 | CD4 | 12p13.31 | CD4 molecule | protein-coding |
| 921 | CD5 | 11q12.2 | CD5 molecule | protein-coding |
| 9212 | AURKB | 17p13.1 | aurora kinase B | protein-coding |
| 9214 | FCMR | 1q32.1 | Fc fragment of IgM receptor | protein-coding |
| 92140 | MTDH | 8q22.1 | metadherin | protein-coding |
| 92241 | RCSD1 | 1q24.2 | RCSD domain containing 1 | protein-coding |
| 9235 | IL32 | 16p13.3 | interleukin 32 | protein-coding |
| 924 | CD7 | 17q25.3 | CD7 molecule | protein-coding |
| 925 | CD8A | 2p11.2 | CD8a molecule | protein-coding |
| 92521 | SPECC1 | 17p11.2 | sperm antigen with calponin homology and coiled-coil domains 1 | protein-coding |
| 92579 | G6PC3 | 17q21.31 | glucose-6-phosphatase catalytic subunit 3 | protein-coding |
| 9275 | BCL7B | 7q11.23 | BCL tumor suppressor 7B | protein-coding |
| 928 | CD9 | 12p13.31 | CD9 molecule | protein-coding |
| 929 | CD14 | 5q31.3 | CD14 molecule | protein-coding |
| 92912 | UBE2Q2 | 15q24.2 | ubiquitin conjugating enzyme E2 Q2 | protein-coding |
| 930 | CD19 | 16p11.2 | CD19 molecule | protein-coding |
| 9308 | CD83 | 6p23 | CD83 molecule | protein-coding |
| 931 | MS4A1 | 11q12.2 | membrane spanning 4-domains A1 | protein-coding |
| 9314 | KLF4 | 9q31.2 | Kruppel like factor 4 | protein-coding |
| 9320 | TRIP12 | 2q36.3 | thyroid hormone receptor interactor 12 | protein-coding |
| 9322 | TRIP10 | 19p13.3 | thyroid hormone receptor interactor 10 | protein-coding |
| 933 | CD22 | 19q13.12 | CD22 molecule | protein-coding |
| 9332 | CD163 | 12p13.31 | CD163 molecule | protein-coding |
| 9334 | B4GALT5 | 20q13.13 | beta-1,4-galactosyltransferase 5 | protein-coding |
| 9353 | SLIT2 | 4p15.31 | slit guidance ligand 2 | protein-coding |
| 9355 | LHX2 | 9q33.3 | LIM homeobox 2 | protein-coding |
| 9370 | ADIPOQ | 3q27.3 | adiponectin, C1Q and collagen domain containing | protein-coding |
| 9376 | SLC22A8 | 11q12.3 | solute carrier family 22 member 8 | protein-coding |
| 9378 | NRXN1 | 2p16.3 | neurexin 1 | protein-coding |
| 939 | CD27 | 12p13.31 | CD27 molecule | protein-coding |
| 94025 | MUC16 | 19p13.2 | mucin 16, cell surface associated | protein-coding |
| 9404 | LPXN | 11q12.1 | leupaxin | protein-coding |
| 941 | CD80 | 3q13.33 | CD80 molecule | protein-coding |
| 942 | CD86 | 3q13.33 | CD86 molecule | protein-coding |
| 9429 | ABCG2 | 4q22.1 | ATP binding cassette subfamily G member 2 (Junior blood group) | protein-coding |
| 943 | TNFRSF8 | 1p36.22 | TNF receptor superfamily member 8 | protein-coding |
| 944 | TNFSF8 | 9q32-q33.1 | tumor necrosis factor superfamily member 8 | protein-coding |
| 9446 | GSTO1 | 10q25.1 | glutathione S-transferase omega 1 | protein-coding |
| 945 | CD33 | 19q13.41 | CD33 molecule | protein-coding |
| 9454 | HOMER3 | 19p13.11 | homer scaffolding protein 3 | protein-coding |
| 947 | CD34 | 1q32.2 | CD34 molecule | protein-coding |
| 9474 | ATG5 | 6q21 | autophagy related 5 | protein-coding |
| 948 | CD36 | 7q21.11 | CD36 molecule | protein-coding |
| 951 | CD37 | 19q13.33 | CD37 molecule | protein-coding |
| 9516 | LITAF | 16p13.13 | lipopolysaccharide induced TNF factor | protein-coding |
| 952 | CD38 | 4p15.32 | CD38 molecule | protein-coding |
| 953 | ENTPD1 | 10q24.1 | ectonucleoside triphosphate diphosphohydrolase 1 | protein-coding |
| 9530 | BAG4 | 8p11.23 | BCL2 associated athanogene 4 | protein-coding |
| 9531 | BAG3 | 10q26.11 | BCL2 associated athanogene 3 | protein-coding |
| 9577 | BRE | 2p23.2 | brain and reproductive organ-expressed (TNFRSF1A modulator) | protein-coding |
| 958 | CD40 | 20q13.12 | CD40 molecule | protein-coding |
| 9582 | APOBEC3B | 22q13.1 | apolipoprotein B mRNA editing enzyme catalytic subunit 3B | protein-coding |
| 9589 | WTAP | 6q25.3 | Wilms tumor 1 associated protein | protein-coding |
| 959 | CD40LG | Xq26.3 | CD40 ligand | protein-coding |
| 9590 | AKAP12 | 6q25.1 | A-kinase anchoring protein 12 | protein-coding |
| 960 | CD44 | 11p13 | CD44 molecule (Indian blood group) | protein-coding |
| 961 | CD47 | 3q13.12 | CD47 molecule | protein-coding |
| 9638 | FEZ1 | 11q24.2 | fasciculation and elongation protein zeta 1 | protein-coding |
| 966 | CD59 | 11p13 | CD59 molecule | protein-coding |
| 969 | CD69 | 12p13.31 | CD69 molecule | protein-coding |
| 970 | CD70 | 19p13.3 | CD70 molecule | protein-coding |
| 9703 | KIAA0100 | 17q11.2 | KIAA0100 | protein-coding |
| 972 | CD74 | 5q33.1 | CD74 molecule | protein-coding |
| 973 | CD79A | 19q13.2 | CD79a molecule | protein-coding |
| 9748 | SLK | 10q24.33-q25.1 | STE20 like kinase | protein-coding |
| 975 | CD81 | 11p15.5 | CD81 molecule | protein-coding |
| 9757 | KMT2B | 19q13.12 | lysine methyltransferase 2B | protein-coding |
| 9759 | HDAC4 | 2q37.3 | histone deacetylase 4 | protein-coding |
| 978 | CDA | 1p36.12 | cytidine deaminase | protein-coding |
| 9826 | ARHGEF11 | 1q23.1 | Rho guanine nucleotide exchange factor 11 | protein-coding |
| 983 | CDK1 | 10q21.2 | cyclin dependent kinase 1 | protein-coding |
| 9833 | MELK | 9p13.2 | maternal embryonic leucine zipper kinase | protein-coding |
| 9846 | GAB2 | 11q14.1 | GRB2 associated binding protein 2 | protein-coding |
| 9873 | FCHSD2 | 11q13.4 | FCH and double SH3 domains 2 | protein-coding |
| 991 | CDC20 | 1p34.2 | cell division cycle 20 | protein-coding |
| 993 | CDC25A | 3p21.31 | cell division cycle 25A | protein-coding |
| 9935 | MAFB | 20q12 | MAF bZIP transcription factor B | protein-coding |
| 994 | CDC25B | 20p13 | cell division cycle 25B | protein-coding |
| 995 | CDC25C | 5q31.2 | cell division cycle 25C | protein-coding |
| 9961 | MVP | 16p11.2 | major vault protein | protein-coding |
| 998 | CDC42 | 1p36.12 | cell division cycle 42 | protein-coding |
| 999 | CDH1 | 16q22.1 | cadherin 1 | protein-coding |
| 9994 | CASP8AP2 | 6q15 | caspase 8 associated protein 2 | protein-coding |
